# Antibacterial smart hydrogels: New hope for infectious wound management

**DOI:** 10.1016/j.mtbio.2022.100499

**Published:** 2022-11-19

**Authors:** Zahra Aliakbar Ahovan, Zahra Esmaeili, Behnaz Sadat Eftekhari, Sadjad Khosravimelal, Morteza Alehosseini, Gorka Orive, Alireza Dolatshahi-Pirouz, Narendra Pal Singh Chauhan, Paul A. Janmey, Ali Hashemi, Subhas C. Kundu, Mazaher Gholipourmalekabadi

**Affiliations:** a Department of Microbiology, School of Medicine, Shahid Beheshti University of Medical Sciences, Tehran, Iran; b Cellular and Molecular Research Centre, Iran University of Medical Sciences, Tehran, Iran; c Department of Medical Biotechnology, Faculty of Allied Medicine, Iran University of Medical Sciences, Tehran, Iran; d Bioengineering Department, University of Pennsylvania, Philadelphia, USA; e Department of Materials Engineering, Isfahan University of Technology, Isfahan 84156-83111, Iran; f NanoBioCel Research Group, School of Pharmacy, University of the Basque Country (UPV/EHU), Vitoria-Gasteiz, Spain; g Biomedical Research Networking Centre in Bioengineering, Biomaterials and Nanomedicine (CIBER-BBN). Vitoria-Gasteiz, Spain; h University Institute for Regenerative Medicine and Oral Implantology - UIRMI (UPV/EHU-Fundación Eduardo Anitua). Vitoria-Gasteiz, Spain; i Bioaraba, NanoBioCel Research Group, Vitoria-Gasteiz, Spain; j Singapore Eye Research Institute, The Academia, 20 College Road, Discovery Tower, Singapore; k Department of Health Technology, Technical University of Denmark, 2800 Kgs. Lyngby, Denmark; l Department of Chemistry, Faculty of Science, Bhupal Nobles' University, Udaipur, Rajasthan, India; m 3Bs Research Group, I3Bs - Research Institute on Biomaterials, Biodegradable and Biomimetics, Headquarters of the European Institute of Excellence on Tissue Engineering and Regenerative Medicine, University of Minho, AvePark, Guimaraes, Portugal; n Department of Tissue Engineering & Regenerative Medicine, Faculty of Advanced Technologies in Medicine, Iran University of Medical Sciences, Tehran, Iran

**Keywords:** Hydrogels, Thermo-sensitive, Thermo-responsive, Wound infections, Burn wounds, Drug delivery

## Abstract

Millions of people die annually due to uncured wound infections. Healthcare systems incur high costs to treat wound infections. Tt is predicted to become more challenging due to the rise of multidrug-resistant conditions. During the last decades, smart antibacterial hydrogels could attract attention as a promising solution, especially for skin wound infections. These antibacterial hydrogels are termed ‘smart’ due to their response to specific physical and chemical environmental stimuli. To deliver different drugs to particular sites in a controlled manner, various types of crosslinking strategies are used in the manufacturing process. Smart hydrogels are designed to provide antimicrobial agents to the infected sites or are built from polymers with inherent disinfectant properties. This paper aims to critically review recent pre-clinical and clinical advances in using smart hydrogels against skin wound infections and propose the next best thing for future trends. For this purpose, an introduction to skin wound healing and disease is presented and intelligent hydrogels responding to different stimuli are introduced. Finally, the most promising investigations are discussed in their related sections. These studies can pave the way for producing new biomaterials with clinical applications.

## Graphical abstract

**Figure:**
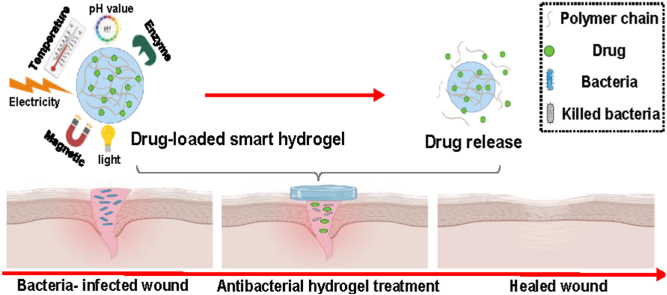

## Abbreviations

**Abbreviations-Definitions**
(Ag NPs): Silver Nanoparticles
(ANAs): Ag Nanoparticle Assemblies
(AA): Acrylic acid
(AL): Alginate
(AMD): AA-co-MADA-co-DMAEMA
(AADs): Active adhesive dressings
(ADA): Oxidized alginate
(AMP): Antimicrobial peptide
(Arg-PEUU/CS-GMA): Arginine-based poly (ester urea urethane) (Arg-PEUU), glycidyl methacrylate-modified chitosan
(AuNPs): gold nanoparticles
(ADH): Adipic acid dihydrazide
(AM): Amikacin
(ABWG): Antibiofilm surfactant wound gel
(bFGF): Basic fibroblast growth factor
(BTDA): 3,30,4,40-benzophenone tetracarboxylic dianhydride
(BA): Phenylboronic acid
(brZnO): Fusiform-like ZnO
(CMC): Carboxymethyl chitosan
(CSDP): Carboxymethyl chitosan and sodium alginate
(CMC): Catechol-modified methacryloyl chitosan
(CTZ): Carboxylated agarose/Tannic acid/Zinc salt
(Cu NPs): Cu nanoparticles
(CMCS): Carboxymethyl chitosan
(CHA): Chlorhexidine acetate
(CA): Citric acid
(CEC): *N*-carboxyethyl chitosan
(CHG): Chlorhexidine Gluconate
(CW): Chitin whisker
(CMCS NPs): Carboxymethyl chitosan nanoparticles
(CSG-PEG): polyethylene glycol monomethyl ether modified glycidyl methacrylate functionalized chitosan
(Cip): Ciprofloxacin
(Cur): Curcumin
(CHX): Chlorhexidine diacetate
(CS): Camellia sinensis
(CeONs): Cerium oxide nanoparticles
(CB-Dex): Carboxybetaine dextran
(CS DABC): Chitosan dialdehyde bacterial cellulose
(CS, GG): Chitosan/Guar Gum
(CNF): Cellulose nanofibers
(CSA): Cationic Steroid Antimicrobial
(COF): Covalent organic framework
(DEG): Diethylene glycol
(DMAEMA): 2-(dimethylamino) ethyl methacrylate
(DN): Double-network
(DMA): Methacrylamide dopamine
(DA): Dopamine
(DG): Dipotassium glycyrrhizinate
(OCMCDA): Dopamine-grafted oxidized carboxymethyl cellulose
(ECM): Extracellular matrix
(EPL): Epsilon-poly-l-lysine
(EPGF): Epidermal growth factor
(EGF): Epidermal growth factors
(EBS): Ebselen
(FGF): Fibroblast growth factor
(Fe3+): Ferric ions
(FA): Folic acid
(Gel-Cip): Ciprofloxacin-loaded polydopamine NPs and glycol chitosan
(GS): Gentamicin sulfate
(GC): Glycol chitosan
(GelMA): Gelatin methacryloyl
(Gel-MA/BACA-Cu NPs): Methacrylate-modified gelatin and N, *N*-bis(acryloyl)cystamine-chelated Cu nanoparticles
(Gel): Gelatin
(GMA): Glycidyl methacrylate
(Gel-Cat): Catechol-modified gelatin
(GTDA/chitosan/CNT): Gelatin-grafted-dopamine and polydopamine-coated carbon nanotubes
(HFFs): Human foreskin fibroblast cell line
(HRP): Horseradish peroxidase
(HOCL): Hypochlorous acid
(HPMC): Hydroxypropyl methylcellulose
(HA): Hyaluronic acid

**(HPMC) Hydroxypropyl methylcellulose**
(HBC): Hydroxybutyl chitosan
(HEMA): 2-hydroxyethyl methacrylate
(HPCH): Hydroxypropyl chitin
(HFBS): Human dermal fibroblasts
(HA–PBA): Phenylboronic acid-modified hyaluronic acid
(HPCS): Hydroxypropyl chitosan
(HAOs): Hyaluronan oligosaccharides
(HA-CHO): Oxidized hyaluronate
(HCCP): Hexachlorocyclic triphosphonitrile
(rhFGF-2): Human fibroblast growth factor 2 FGF-2
(HS): Hidradenitis Suppurativa
(IA): Itaconic acid
(Irgel): Iranian hydrogel sheet
(JIS): Japanese industrial standards
(KGF): Keratinocyte growth factor
(KGM): Konjac glucomannan
(LCST): Low critical solution temperature
(LAP-DMA): Laponite-dopamine
(MUG): 4-methyllumbelliferyl ẞ-D- glucuronide
(MMPs): Matrix metalloproteinase
(MRC-5): Medical Research Council cell strain 5
(MC): Methacryloyl chitosan
(MSSA): Methicillin-sensitive *Staphylococcus aureus*
(MDR): Multi-drug resistance pathogens
(MADA): Methacrylamide dopamine
(MRSA): Methicillin-resistant *Staphylococcus aureus*
(ML-D): Multi-layer with drug
(ML): Multi-layer
(mXG): Modified xyloglucan
(MC): Methacryloyl chitosan
(MUD): Methylumbelliferyl-α-Dglucopyranoside acid
(MeTro): Methacryloyl tropoelastin
(M-Arg): Methacrylate arginine
(Met): Metformin
(M-WELQK-M): Methacrylate-WELQK-methacrylate
(N, O-CMC/OCS): N, O-carboxymethyl chitosan/oxidized chondroitin sulfate
(NPs): Nano-particles
(NIR): Near-infrared
(NIPAAm): *N*-isopropyl acrylamide
(NMS): Neomycin sulfate
(NIM): Nimesulide
(NEO): Neomycin
(Nap): Naproxen
(OD): Oxidized dextran
(OSEA): Oxidized salep and ethylene diamine-modified salep (SaHEA) chains
(OHA-AT): Oxidized hyaluronic acid-graft-aniline tetramer
(OCMC): Oxidized carboxymethyl cellulose
(Octenisept®, OCT): octenidine dihydrochloride and 2-phenoxyethanol
(OKGM): Oxidized Konjac glucomannan
(PCA): Polyvinyl alcohol/chitosan/silver nanoparticle
(PNPG): 4-nitrophenyl ẞ-D- glucuronide
(PDGF): Platelet-derived growth factor
(PVA): Polyvinyl alcohol
(PAM): Polyacrylamide
(PVP): Polyvinylpyrrolidone
(ε-PL): ε-polylysine
(PILs): Polymerized ionic liquids
(PEGS-FA): Poly (glycerol sebacate) grafted 4-formylbenzoic acid
(PNIPAAm): Poly (*N*-isopropylacrylamide
(PN): Pomegranate
(PAS): poly (AAm-co-SVBA)
(PF127): Pluronic® F127
(PACT): Photodynamic antibacterial chemotherapy
(PAA): Poly acrylic acid
(PEG): Poly ethylene glycol
(PEGD): Poly ethylene glycol diacrylate
(PEI): Poly (ethylene imine)
(PDA@ Ag NPs/CPHs): Poly dopamine decorated silver nanoparticles/conductive polymer-based Hydrogels
(PDEAEMA): Poly (diethylaminoethyl methacrylate)
(PDMAEMA): Poly (dimethylaminoethyl methacrylate)
(PMAA): Poly (methacrylic acid)
(γ-PGA): γ-polyglutamic acid
(PCBAA-1-C2 SA): [poly(N-1-(ethoxycarbonylmethyl)-*N*- (3-acryloylamino-propyl)-N, *N*-dimethyl ammonium salicylate)]
(PEP): Poly (*N*-isopropylacrylamide166-co-n-butyl acrylate9)-poly (ethylene glycol)-poly (*N*-isopropylacrylamide166-co-n-butyl acrylate9) copolymer (P(NIPAM166-co-nBA9)-PEG-P(NIPAM166-conBA9)
(PPY@PDA NWs): polydopamine-coated polypyrrole nanowires
(PDA@Ag NPs): polydopamine decorated silver nanoparticles
(PCDQ): Phosphatidylcholine dihydroquercetin
(PL): Pluronic F127
(PDEAAM): Poly (N, *N*-diethylacrylamide)
(PNIPA and PNIPAM): Poly (*N*-isopropylacrylamide)
(PEO): Polyethylene Oxide
(PDA/Cu-CS): Polydopamine and copper-doped calcium silicate ceramic
(PNIPAM): poly(*N*-isopropylacrylamide)
(PLGA): Poly (lactic-co-glycolic acid)
(PGMF): Punica granatum peel ethanolic extract
(QCS): Quaternized chitosan
(QCSP): Quaternized chitosan polyaniline
(QC): Quaternized chitosan
(QCS-M-PAM): Quaternized chitosan-Matrigel-polyacrylamide
(QCS-Polyaniline): Quaternized Chitosan-graft-Polyaniline
QP-(DMAPMA): Quaternized *N*-[3(Dimethylamino) propyl] methacrylamide
(ROS): Reactive Oxygen Species
(RAFT): Reversible addition-fragmentation chain transfer
(rGO): Reduced graphene oxide
(SD): Sprague Dawley
(SplB): Serine protease-like B enzyme proteins
(SA): Salicylate
(SNP-Cy3/Cy5): Silica nanoparticles modified with Cyanin 3 and 5
(SA): Sodium Alginate
(SPR): Surface plasma resonance
(SB-Dex): Sulfobetaine dextran
(SEA): Sodium ethylene glycol alginate
(SNC): Silver nanocomposite
(SVF): Stromal vascular fraction
(SVBA): zwitterion poly[3-(dimethyl(4-vinylbenzyl) ammonium) propyl sulfonate
(SS): Sisomicin sulfate
(GSNO): S-nitrosoglutathione
(TA): Tannic acid
(3T-CHO): Trithiophene aldehyde
(3 ​TT): Tryptophan-modified trithiophene aldehyde
(Try): Trypsin
(TEMPO): 2,2,6,6-tetramethylpiperidine-1-oxyl-doped
(TOB): Tobramycin
(TEOS): Tetraethoxysilane
(UCNP): Up-conversion nanoparticles
(UCST): Upper critical solution temperature
(VL): Visible light
(VCM): Vancomycin
(VBIMBr): 1-vinyl-3-butylimidazolium
(VEGF): Vascular endothelial growth factor
(WHO): World Health Organization
(XDR): Extensively drug-resistant
(ZOI): Zone of inhibition
(ZPFSA): Piezoelectric hydrogel scaffold
(ZnO NPs): Zinc oxide nanoparticles

## 1 Introduction

Skin is the largest organ in the human body and plays a pivotal function in defending it from external damage (thermal and physical) and invasive microorganisms [1]. When the integrity and function of this organ are interrupted, skin wounds occur [2]. Wounds are divided into two groups: acute ones, such as burns, surgical wounds, and traumas and chronic ones, like venous legs, diabetes, and pressure ulcers [3,4]. According to the World Health Organization (WHO), around 265,000 deaths are reported annually just due to wounds created by burns [5]. Over and above that, microbial infections on the injured site are another significant problem, as they prolong the healing process of wounds and can be lethal for patients [5,6]. Additionally, overuse of antibiotics leads to increased antibiotic resistance while limiting the efficacy of therapeutic modalities [7,8]. Also, due to the widespread use of systemic antibiotics for local infections, antibiotic resistance has become an urgent global concern that requires effective treatments [9,10]. Wound management is mainly concerned with optimizing wound healing by preventing bacterial colonization. Over the last few decades, a great deal of effort has been put into developing dressings with antibacterial properties that can stimulate wound healing [11]. Thus, wound dressings must possess antibacterial or anti-fouling activities to prevent infections at the wound site. For instance, by controlling the adsorption of fibrinogen and its subsequent transformation into a fibrin gel, they can do wonders in terms of bypassing a potential haven for bacteria and unwanted inflammatory responses [12]. Applying these dressings that either contain antibacterial agents or are made from polymers with inherent antimicrobial properties leads to a reduced rate of antibacterial resistance [1]. Hydrogels have recently become more attractive among different types of novel antibacterial wound dressings due to their intrinsic drug delivery capacity [13]. The significant merit of hydrogels is that they are composed of hydrophilic chains, which maintain moisture around the wound site. A smart hydrogel, on the other hand, is, in simple terms, defined as a 3D structure that can significantly change its size or mechanical stability depending on environmental conditions and is among the most investigated types of biomaterials in the field [14]. This so-called smart hydrogels can control drug release and display sensitivity to stimuli such as light, pressure, magnetic fields, and temperature, plus chemical and biological stimuli such as enzymes and ROS [15,16]. Several hydrogels have already been developed as drug delivery systems and are capable of releasing antibacterial agents depending on environmental conditions in order to prevent wound infection [17]. Importantly, the main advantage of hydrogels for wound care is intimately related to their hydrophilic chains, which in turn maintain moisture around the wound site. Here, we will review some of these hydrogel systems and explore their characteristics while simultaneously discussing recent advancements in the manufacturing of smart hydrogels, especially their wound healing applications.

## 2 Skin and wound structure

**Fig. 1:**
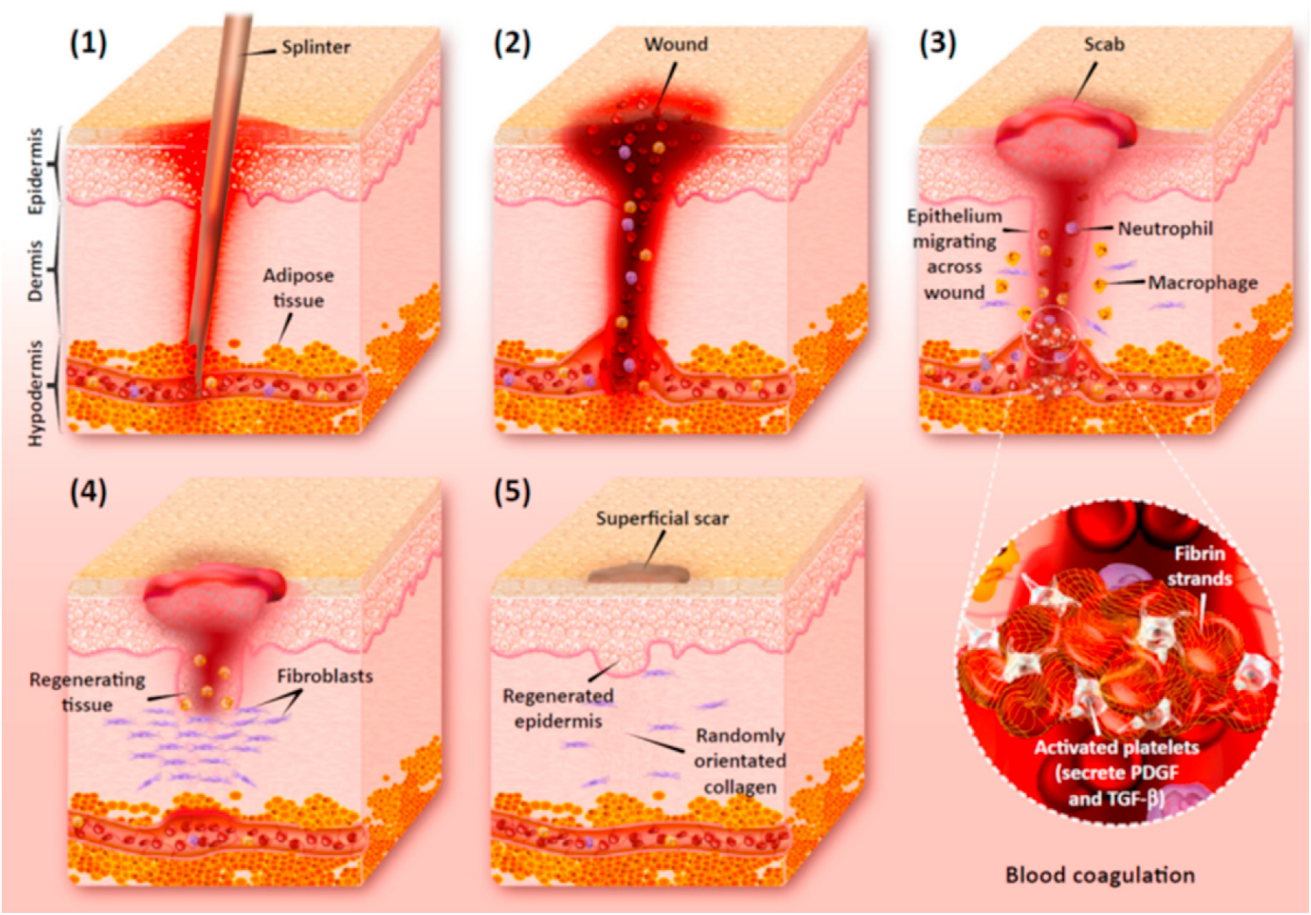
The structure of human skin contains three essential layers: the epidermis, the dermis, and the hypodermis (1); hemostasis, inflammation, proliferation, and remodeling are the four critical steps of the wound healing process. During hemostasis, platelets stick to one another, inducing clot formation (2); then, the inflammatory phase begins with the recruitment of immune cells, where they phagocyte dead cells, bacteria, and other pathogens or debris (3). At the proliferation stage, epithelialization occurs by replacing dead cells with epithelial cells, thus activating the angiogenesis process (4). Finally, tissue is regenerated by degrading the excessive collagen (5). Reproduced with permission [34]. Copyright 2018, Elsevier.

As a vital multi-layer organ, skin comprises three layers: epidermis, dermis, and subcutaneous tissue [18,19]. It acts as an interface between internal and external organs, regulates body temperature, and protects the body from injury and microbial invasion [20]. Damage to the physiological structure of the skin defines a wound, which can be extended to other tissues, including subcutaneous tissues and nerves [[21], [22], [23], [24]]. Dermal wounds are categorized into two main groups: acute and chronic. An accident, surgical injury, or burn causes an acute wound that usually leads to tissue damage. The skin recovers within an 8- to 12-week period with a minimum scar formation, depending on the scale of the injury to its layers [21]. In chronic wounds like diabetes and pressure ulcers, a considerable amount of tissue is lost. Usually, the duration of treatment for this type of wound is longer than 12 weeks [3,25]. Chronic wounds are a place to colonize some bacterial pathogens, including *Pseudomonas aeruginosa*, *Staphylococcus epidermis*, and *Staphylococcus aureus*. [17] with potentially devastating outcomes for the patient*.* According to statistics from the United States, more than 2% of the U.S. population complains about chronic wounds. More than 20 billion dollars is spent on treating these patients annually [26]. Hemostasis, inflammation, proliferation, and remodeling are the four categories that constitute a normal wound healing process (Fig. 1) [27,28]. Platelets are released at the site after an injury and adhere to the damaged blood vessel walls. The inflammatory response begins with the active absorption of neutrophils and inflammatory cytokines released from platelets, endothelial cells, etc. [29] Then, epithelialization occurs 48 ​h after the beginning of the wound healing process and continues for up to 14 days post-injury [30]. The remodeling phase occurs when collagen III is degraded and the synthesis of collagen I is increased. About two weeks post-injury, the wound becomes smaller, and a small scar forms at the site [31]. Also, many factors such as cytokines, the extracellular matrix (ECM), macrophages, neutrophils, fibroblasts, epidermal growth factors (EGFs), fibroblast growth factor (FGFs), and platelet-derived growth factors (PDGFs) participate in the process [32]. Environmental conditions around the wound site, such as humidity, infection, and diabetes, have been shown to affect the normal healing process, as well [33].

## 3 Post-wound infection

**Fig. 2:**
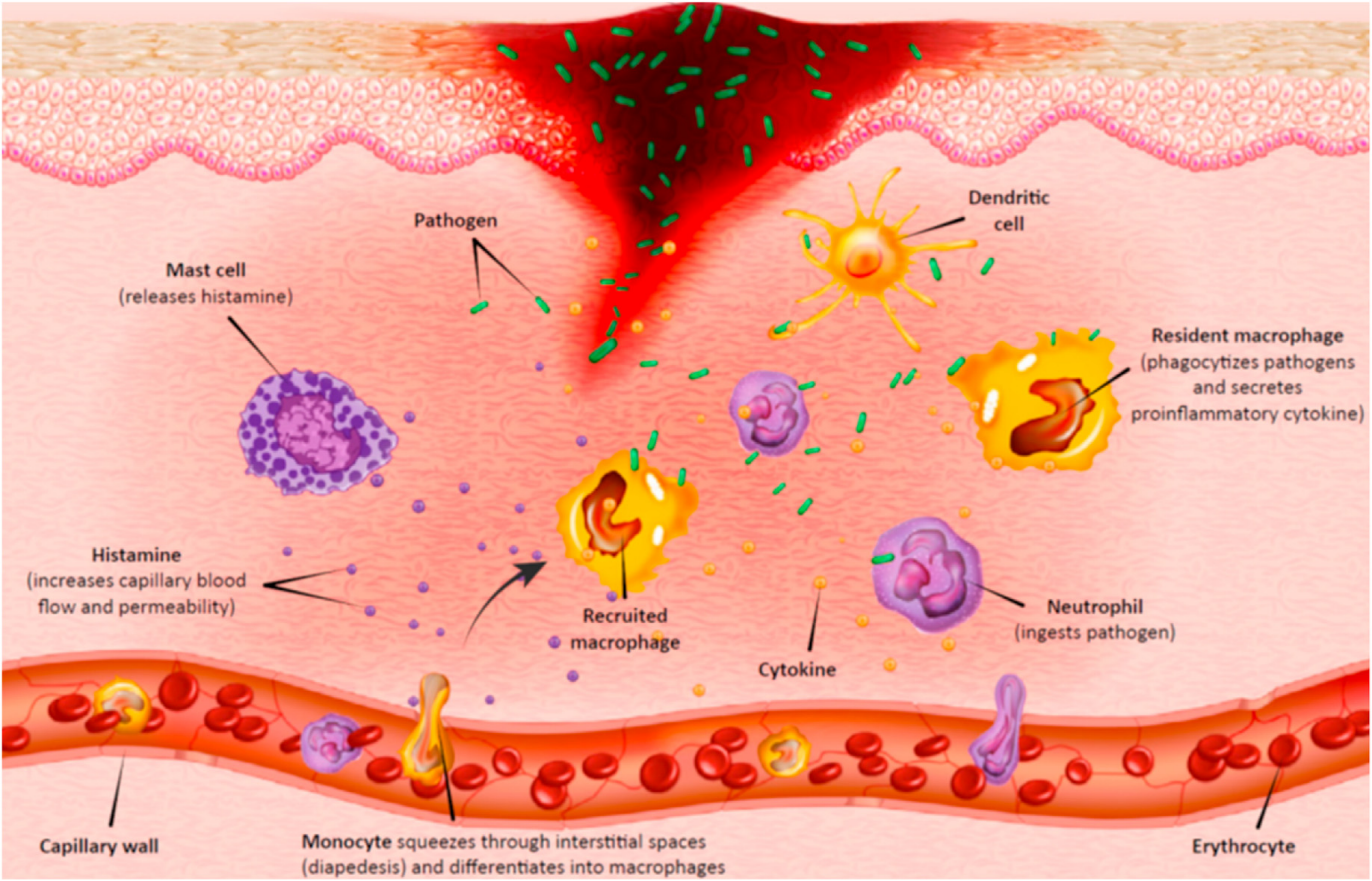
Wound infection emerges by the presence of bacterial colonies on the wound site. Immune cells like macrophages and neutrophils are responsible for eliminating pathogens at the wound bed. Reproduced with permission [34]. Copyright 2018, Elsevier.

Wound infection is a severe concern in the healthcare system. Indeed, opportunistic pathogens can invade, colonize, and proliferate at the wound site in various types of wounds, like burns and traumas, which may cause infection (Fig. 2) [[35], [36], [37]]. A prolonged wound healing process and, in some cases, disability and death are the results of infected wounds [38]. *P. aeruginosa*, *S. aureus*, *S. epidermidis*, *Escherichia coli*, and *Acinetobacter baumannii* are some of the leading pathogens causing wound infections. Due to the misuse of antibiotics, some multi-drug resistant (MDR) strains have emerged in recent years [39,40]. For example, increased resistance to many antibiotic groups like carbapenems, aminoglycosides, and quinolones have been reported in *P. aeruginosa* isolates [41]. According to a CDC report, in the United States, about 13% of 51,000 cases infected by *P. aeruginosa* are MDR isolates, which result in more than 400 deaths annually. Therefore, effective treatment with antibiotics has been limited; in many cases, they cannot prevent infections completely [6]. For preventing antibiotic resistance and reducing mortality, it is essential to design new strategies and technologies that use antibiotics or antibacterial agents. For instance, small bioactive proteins like antimicrobial peptides (AMPs) have become the first-line defense against pathogens [42]. AMPs have several antimicrobial mechanisms such as their effect on the cytoplasm, the endocytosis of the bacterial plasma membrane, or interrupting their cell cycle via intracellular activities [[43], [44], [45]]. Another strategy is to use nanoparticles (NPs) like silver nanoparticles (AgNPs), gold nanoparticles (AuNPs) [46,47], and copper nanoparticles (CuNPs) [48], which are recommended as wound therapies to prevent the growth of biofilms on wound infections [49,50]. The primary mechanism of NPs is that they can penetrate the bacterial cell walls, then positive charges of NPs are linked to negatively-charged sectors at the surfaces of bacteria. As a result, hydrophobic interactions can lead to holes in bacteria's surfaces. Also, they adversely impact the proton efflux pumps and subsequently, with a modification in the pH range, destroy the membrane's surface charge [50]. Essential oils and honey are other well-known examples of materials in wound care [51,52]. Honey possesses a naturally acidic pH, provoking macrophages to reduce bacteria and biofilm levels. Essential oils like tea tree oil and chamomile oil have antibacterial activity and negatively impact the integration between bacterial cells and cellular membranes [53]. natural antibacterial agents more effectively reduce infections by mechanisms to which the pathogen cannot develop resistance by mutation as readily as they can to specific drugs [54]. In the past few decades, novel wound dressings with drug delivery capacity to the target sites have been developed to prevent bacterial infections and enhance wound healing [55].

## 4 Dermal wound dressing

As the threat of wound infections spreads, optimizing the antibiotic or antibacterial agents has become essential [56,57]. Recently, the traditional ways of using antibiotics, like local or systemic therapies, have lost their entire bactericidal potential [58]. In these ways, antimicrobial resistance and risks of adverse side effects may rise further due to insufficient antibiotic dosage at the site of wounds [59,60]. Gauze, cotton, and bandages have been used for centuries as conventional wound dressings to overcome the health issue linked to wound infections. Many disadvantages include keeping the wound area dry, maintaining good adhesion to the underlying tissue, and assuring an easy bandage removal with minimal patient discomfort [21]. Today, novel wound dressings are prepared in various forms adapting to the type of wound, treatment duration, injury condition, and infection prevention purposes [21,[61], [62], [63]]. An ideal dressing for healing wounds requires some essential properties, such as strong mechanical properties and delivering a sufficiently strong barrier against microorganisms, while keeping the damaged site moistened and promoting angiogenesis [64]. In addition, improving suitable local delivery systems for antibiotics has attracted lots of attention [65]. An optimal drug carrier can keep a higher antibiotic dosage in the wound site for a long time, reduce the adverse effects of systematic administration in the host tissues, and decline the standard doses of antibiotics, which can enhance the race of antibiotic resistance. The sustained release of antibacterial agents is significantly vital in the process of wound healing [50]. Polymers with inherent antibacterial activity like chitosan or modified chitosan or an antibacterial agent such as antibiotics or NPs can be used to prepare various antibacterial wound dressings like hydrogels, foam, nanofibers, and membranes [66,67]. In this direction, hydrogels have multifunctional properties that make them desirable candidates as a novel wound dressing [64]. Thus, a suitable hydrogel should enable the repair of body tissues with the least demand for cell growth, proliferation, and vascularization [68]. However, scaffold degradation should happen through or immediately after the healing process. Also, the suitable hydrogels can be used as a drug delivery system to control the release of antibacterial agents and must possess a high porosity rate and a desirable swelling ability [[69], [70], [71]]. Despite the advantages of hydrogels, some of their limited properties, like mechanical behavior, strength, biocompatibility and stiffness, should be enhanced [72]. Several studies have been conducted to overcome these drawbacks. For instance, using argon micro-plasma as a neutral energy source for cross-linking has replaced the classical cross-linking processes. Another hydrogel-related dilemma is about its non-adherence; they need to be fixed by a secondary dressing, thus they may have low compatibility for moderate or high exudation wounds. The process of sterilization of synthetic hydrogels is complicated due to the proportion of water in these materials that can adversely affect this process [73].

## 5 Hydrogels

**Fig. 3:**
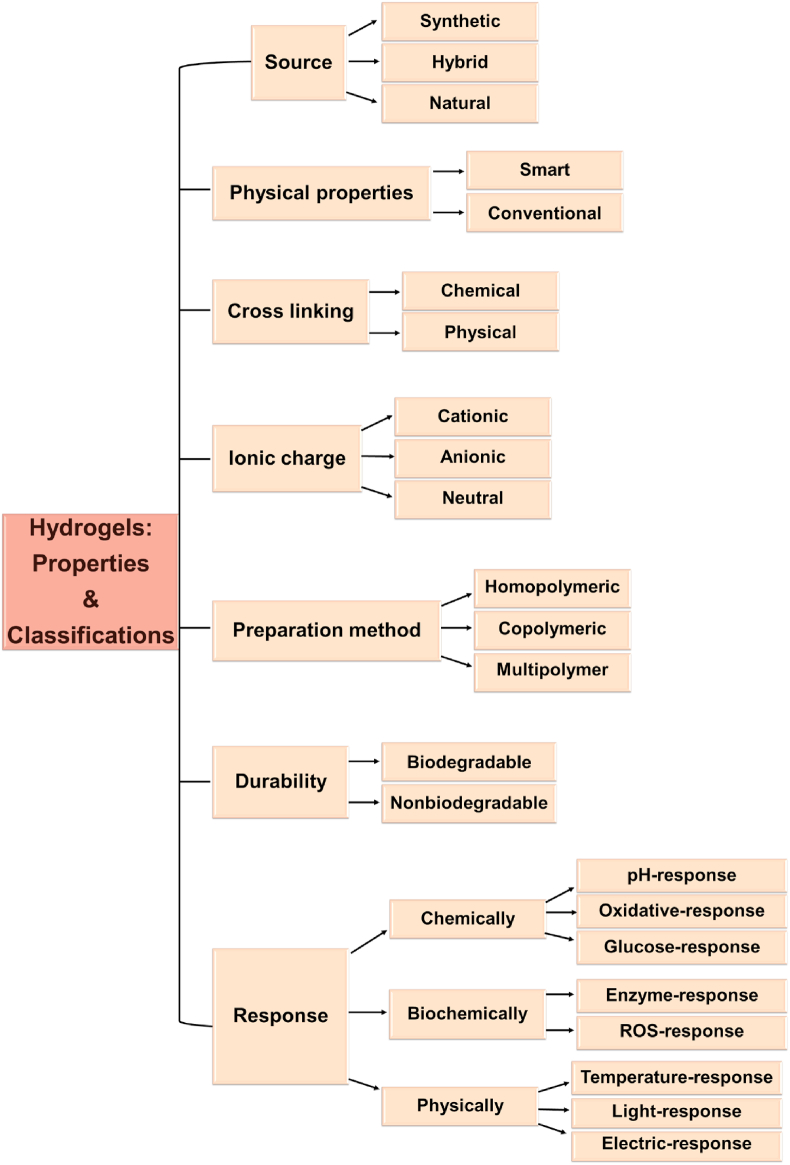
Classification of hydrogels depending on the different factors.

Hydrogels are formed by water-soluble synthetic and/or natural polymers with a dynamic crosslinking composition that imitates the structure and functions of the ECM [74,75]. These biomaterials are attractive candidates for regenerative medicine because of their excellent mechanical properties like elasticity, swelling, and ability to transfer nutrients and waste quickly [76]. For wound healing purposes, a desirable hydrogel must repair tissues and provide an ideal environment for cell proliferation, vascularization, and host integration [77]. Hydrogels also enable good oxygen transfer between the wound site and atmosphere and possess favorable biocompatibility, biodegradability, sufficient swelling capacity to absorb wound exudates, and antibacterial properties; they are non-toxic and non-allergenic [[78], [79], [80]]. In drug delivery systems, hydrogels have a highly porous structure leading to a sustained release of drugs [81]. Hydrogels are divided into many categories. Based on polymer origin, hydrogels are split into three groups. Natural polymers like alginate, dextran, chitosan, pectin, chitin, cellulose, etc., produce highly biocompatible products, as some naturally exist in ECM [[82], [83], [84]]. Matrigel and Fibrinogel are commercially available as natural hydrogels [85,86]. Synthesized polymers such as polyvinyl alcohol (PVA), polyethylene oxide (PEO), and polyethylene glycol (PEG) are less biocompatible than natural hydrogels because of their physical and chemical properties [86,87]. The third group, hybrid hydrogels, is constructed of natural and synthetic polymers. These systems offer high biodegradability and biocompatibility. According to another classification, hydrogels are categorized depending on their cross-linking junctions, such as physical and chemical ones (Fig. 3). The physical ones encompass hydrogen bonds, and electrostatic and hydrophobic interactions, while the chemical ones are primarily made from various covalent bonding schemes [88]. They are also divided into two groups: non-bioactive and bioactive polymers. Despite advancements in using these hydrogels to respond sequentially to pathological and physiological changes that happen within the environment, there are still a number of obstacles to overcome. Thus, studies are designed to develop innovative bioactive hydrogels for biomedical applications to promote better tissue regeneration [89,90].

### 5.1 Smart hydrogels

Smart hydrogels contain specific functionality that increases cell proliferation, migration, differentiation, and blood vessel formation while significantly reducing the wound-healing period [[91], [92], [93]]. Specifically, they are three-dimensional networks constructed using various chemical, physical, and biological cross-linking systems. These crosslinkers enable them to respond to their surrounding environmental stimuli, including physical changes like temperature, light, magnetic and electric fields, and chemical alterations such as pH, ions and particular molecules, enzymes, and antigens. Innovative hydrogel responses include variations in swelling behavior, permeability, network architecture, mechanical properties, and sol-gel transition [[93], [94], [95]]. The following sections of the current review describe such stimuli-sensitive hydrogels for infected wound healing.

#### 5.1.1 pH-sensitive hydrogels

**Fig. 4:**
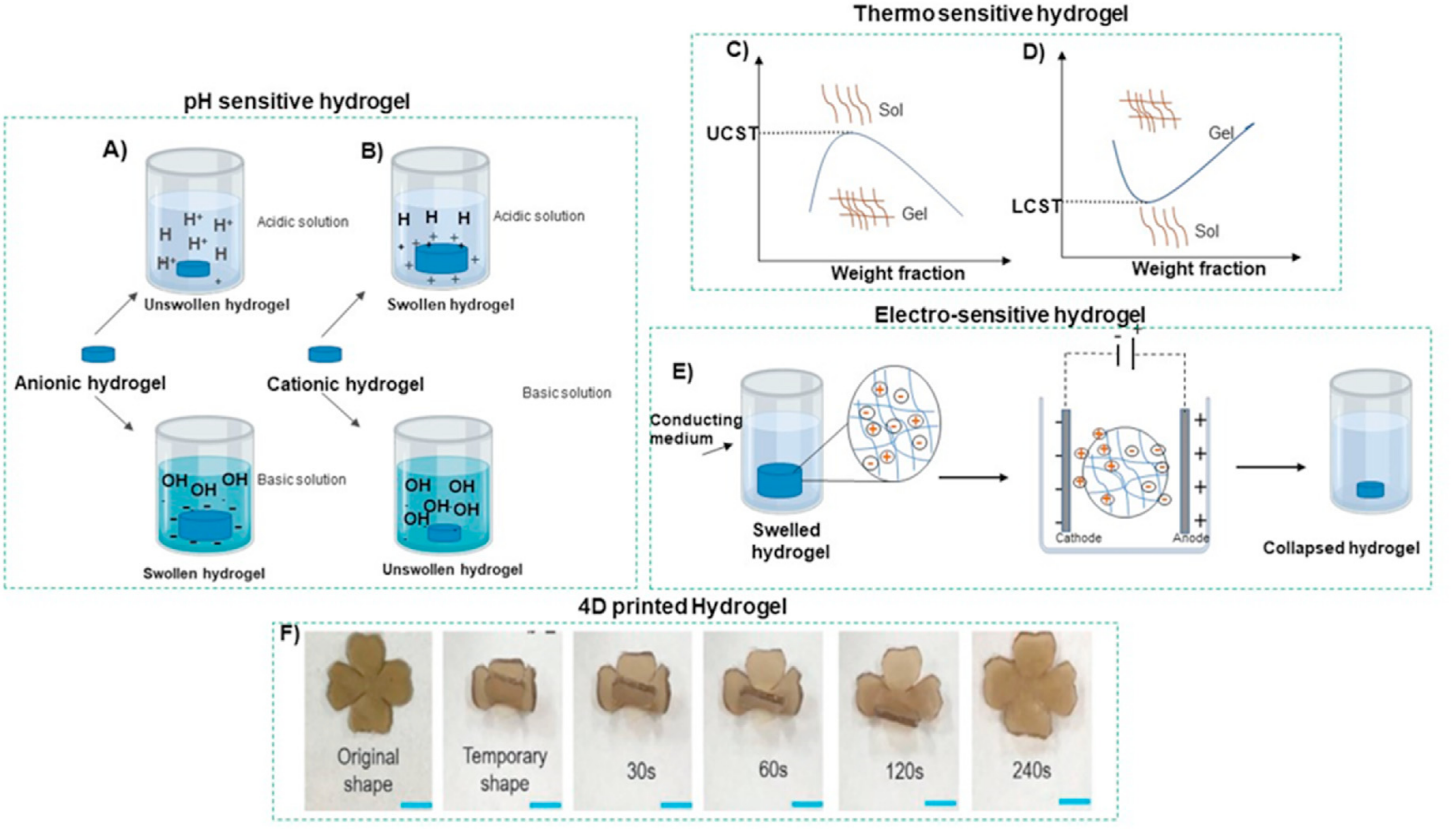
The swelling property of (A) anionic and (B) cationic polymers in response to pH. Curves show the sol-gel transition phenomenon for (C) upper critical solution temperature (LCST) and (D) lower critical solution temperature (UCST) phase transition of thermo-sensitive hydrogels. E) Schematic illustrating the electro-sensitive hydrogels' transition of volume when a voltage is applied. F) the photoresponse shape recovery activity of 4D printed F127DA/PLGA/graphene oxide hydrogel under NIR [96].

pH-sensitive hydrogels are described as “ionic” when containing acidic pendant groups (such as carboxyl acid) or cationic groups (like amine). Some synthetic polymers such as poly (acrylic acid) (PAA), poly (methacrylic acid) (PMAA), polyacrylamide (PAM), poly (diethylaminoethyl methacrylate) (PDEAEMA), and poly (dimethylaminoethyl methacrylate) (PDMAEMA), and several natural ones like alginate (cationic), chitosan (anionic), albumin, and gelatin are designed as pH-responsive gels [97]. These gels' water swelling water capacity depends upon several factors, including hydrophilicity, pH of the external medium, ionic charge, degree of ionization, pKa or pKb values of ionizable groups, and polymer concentration. Among these factors, the nature of ionizable groups, pH, and ion composition is among the main factors determining the properties of smart hydrogels [98]. At low pH, polymers that contain carboxyl groups are unswollen because the acidic group will be protonated, and hydrophobic interactions between the chains of polymer cause volume shrinkage. As the pH increases, the carboxyl groups become highly ionized, resulting in a high internal charge repulsion, which leads to swelling [99]. In contrast to carboxyl-based polymers, the ionization of polybasic polymers will enhance under an acidic environment, and swelling in the overall dimensions of these hydrogels results. Fig. 4(A) and (B) respectively depict the swelling of anionic and cationic responses to pH in hydrogels. Thus, in reaction to the pH of the environment, drug release from these smart hydrogels shows a controlled-release pattern [100].

#### 5.1.2 Thermo-sensitive hydrogels

These polymers are achieved through a synthetic process. A hydrophobic monomer region (e.g., methyl, ethyl, and propyl groups) is coupled to a hydrophilic segment (e.g., carboxyl and amide) to fabricate an amphiphilic polymer [101,102]. The polymeric network will change configuration above a particular temperature, also referred to as the low critical solution temperature (LCST). For instance, as the temperature decreases below the LCST, the structure shrinks by the aggregation of hydrophobic regions (Fig. 4C) [[103], [104], [105]]. Notably, the LCST can be controlled by altering the hydrophobic to the hydrophilic ratio in the polymer layout. Among temperature-responsive polymers, poly (N, *N*-diethylacrylamide) (PDEAAM) is the most commonly applied to fabricate controlled drug delivery systems because its LCST is around body temperature (25–32 ​°C) [106]. Also, some hydrogel structures are formed under a critical temperature, known as the upper critical solution temperature (UCST) (Fig. 4D). These types of polymers, such as poly (acrylic acid) (PAA), poly (n-isopropyl acrylamide), and poly (acrylamide-co-butyl methacrylate), enter an unfolded configuration above the UCST, and thereby their solubility is increased.

#### 5.1.3 Light-sensitive hydrogels

Light-responsive hydrogels are composed of a photo-reactive functional group that can capture photo signals to regulate physical and chemical properties. Some of these hydrogels have been found helpful as ophthalmic drug delivery systems because the light stimulus may be applied accurately and promptly. However, such intelligent hydrogels' slow response time is a severe limitation [107]. Light-responsive hydrogels are divided into two groups: UV- and visible-light-sensitive. The former is prepared by attaching a leuco derivative molecule like bis (4-di- methylamino) phenylmethyl leucocyanide into the polymer structure. Under UV radiation, the triphenylmethane leuco dye is ionized into triphenylmethyl cations, and electrostatic repulsion between photogenerated ions causes photoinduced swelling. Light-active chromophores (azobenzene moieties, e.g., trisodium salt of copper chlorophyllin) can be incorporated into the polymer structure to synthesize a visible light hydrogel (e.g., 488 ​nm) [108,109]. The chromophore can absorb applied light, which is then disintegrated as heat, increasing the hydrogel's local temperature. The temperature increment changes the swelling behavior of thermo-sensitive hydrogel (e.g., poly(*N*-isopropylacrylamide) hydrogels). Several factors, such as the light intensity and the chromophore concentration, determine the amount of temperature increment [110,111].

#### 5.1.4 Electro-sensitive hydrogels

Electric and magnetic field-responsive hydrogels are investigated as polyelectrolyte polymers with a high proportion of ionizable systems, as are pH-sensitive hydrogels [112,113]. Any variations in electric current or fields can cause hydrogels to swell, bend, or shrink. Such electro- and magnetic-responsive materials are typically made from synthetic polymers, including acrylic acid (AA) and PVA, as well as natural polymers like chitosan, alginate, and hyaluronic acid (HA) [114]. When the anode and cathode electrodes are placed in contact with hydrolyzed polyacrylamide hydrogels’ surface, the generated electrical potential collapses the volume of the hydrogels (Fig. 4E). Electro-sensitive hydrogels are utilized for drug delivery controlled by varying the electrical field intensity [115].

#### 5.1.5 Biologically-sensitive hydrogels

Biomolecules (including ions, enzymes, and specific antigens) are used as the crosslinkers for hydrogel systems that are sensitive to the biological environment by being able to become cleaved under one particular biochemical reaction [116]. As an example, an ion-responsive hydrogel, poly (*N*-isopropylacrylamide) (PNIPA) gel, can transform to a collapsed state at a specific concentration of sodium chloride [117,118]. Specific antigen-responsive hydrogels are developed to fabricate biomedical devices that can respond to particular proteins. To this end, specific crosslinking interactions between antigens and antibodies are used to create these smart hydrogels. Antigen-antibody hydrogels' crosslinking density and swelling ratio are reversibly changed according to antigen density. The glucose-sensitive phase-reverse hydrogel is also among some of the most common biological-responsive hydrogels. Other systems are based on bonds that can become cleaved by the families of enzyme matrix metalloproteinases (MMPs) thereby opening pathways for controlled hydrogel degradation [119].

#### 5.1.6 Four-dimensional (4D) printed hydrogels

The rapid development and interdisciplinary research of smart biomaterials and 3-dimensional (3D) bioprinting created 4D bioprinting as the next generation of biofabrication technology. At a 2013 TED Talk, the hypothesis of 4D printing was initially introduced by Skylar Tibbits as 3D printed construct which could undergo architectural changes in a time-dependent manner [120,121]. Compared to 3D printed structures, 4D printing allows for animated designs that can alter their configuration or function with time under specific external stimuli like pH, moisture, heat, and light. In addition, 4D-printed objects can respond to environmental interaction and stimuli with various outputs, including mechanical movements and biological reactions. Thus, the field of 4D printing, still in its infancy, has enticed growing interest from research and industry of various disciplines [122,123]. Among a wide range of materials, novel hydrogels with stimuli-responsiveness have gained more popularity for 4D printed systems because of their large deformability, excellent flexibility, desirable biocompatibility, straightforward manufacturing process, and inexpensiveness compared to metal and ceramics. Some polymers like poly(*N*-isopropylacrylamide) (PNIPAm), poly(*N*,*N*-dimethylacrylamide) (PDMAAm), alginate, hyaluronic acid, and gelatin have been intensively studied as stimuli-response hydrogels for 4D printing applications [124,125]. Moreover, extrusion-based strategies such as direct ink writing (DIW), fused deposition modeling (FDM), and vat polymerization methods like stereolithography (SLA), digital light synthesis (DLS), and digital light processing (DLP) are the most efficient printing techniques for hydrogels [126].

Dai et al. synthesized a double network-shaped memory hydrogel by chemical cross-linking of Pluronic F127 diacrylate macromer (F127DA) and physically blended poly(lactide-co-glycolide) (PLGA) with graphene oxide (GO, for near-infrared (NIR) irradiation conversion to thermal energy as an energy convertor. This hydrogel was fabricated with a 3D-printing technique using UV light polymerization and proposed for using in drug delivery purposes by NIR radiation (Fig. 4F) [96]. Larush and coworkers [127], employed the DLP method to manufacture an oral drug delivery system using acrylic acid monomers, polyethylene glycol diacrylate as the crosslinker, and photoinitiator (2,4,6-trimethylbenzoyl-diphenylphosphine oxide [TPO] nanoparticles). This study showed that the printed object could control the release of drugs with pH and by fine-tuning geometric variables. In summary, recent studies demonstrate that 4D-printed hydrogels could provide the potential to influence the localization and drugs’ release rate. Despite remarkable progress in the 4D printing technique, it is still facing several challenges and opportunities. Firstly, from the perspective of fabrication techniques, the most common printing methods for hydrogels (such as DIW, FDM, DLS, and DLP) have several disadvantages, including low resolution, the demand for expensive equipment, slow printing speed, and limited printing materials. The recent evidence suggests the material jetting and binder jetting methods as potential candidates because of their less restrictive material types and higher production rates [128,129]. Secondly, from the material perspective, the hydrogel response to stimuli is restricted to heat, moisture, and chemical environment. To expand the application of 4D-printed hydrogel devices, it is necessary to explore new types of stimuli. For instance, electrically conductive and stretchable hydrogels possess the potential to be employed as the matrix of electrical- and mechanical-responsive hydrogel composites [130].

## 6 Antibacterial smart hydrogels

Various types of intelligent hydrogels with antimicrobial activities have been created to prevent infection and enhance wound healing [131,132]. For example, smart hydrogels have been utilized as drug delivery systems enabling controlled drug release in response to environmental variables [133]. Recently, they also have been designed as a wound dressing to sustain the release of antibacterial agents and prevent the colonization of pathogens at the wound site [134,135]. Several strategies are used to prepare such smart antibacterial hydrogels. For example, polymers with inherent antibacterial activity like chitosan and/or loaded antibacterial agents such as nanoparticles, polypeptides, and antibiotics have been frequently used.

### 6.1 pH-sensitive antibacterial hydrogels as wound dressings

Different pH-sensitive antibacterial hydrogels have been fabricated as wound dressings to inhibit or reduce infections and promote wound healing. pH-sensitive hydrogels change their swelling ratios dependent on pH changes [136]. Swelling is a unique parameter for the assessment of pH-sensitive hydrogels [137]. Several studies along these lines have reported numerous factors that affect the swelling rate of ionic hydrogels for drug release in physiological conditions, including pore size, ionic charge, cross-linking density, speed of ionization, and ionic stability [133,138]. The pH of the wound site acts as an essential parameter in the wound healing process. The pH of native skin is around 5.5. However, it may vary depending on the wound type, wound healing phase, and infection [139]. Different polymers with ionic functional groups are used to prepare pH-sensitive hydrogels [140]. Protonation of amino groups in cationic polymers like chitosan and poly (ethylene imine) (PEI) leads to swelling at low pH. Anionic hydrogels like carboxymethyl chitosan (CMC), with acidic groups' ionization, lead to swelling at higher pH values [133]. Ninan et al. [139] designed an antibacterial carboxylated agarose/tannic acid/zinc salt (CTZ) pH-sensitive hydrogel. To control the pH of tannic acid release, the zinc ions were used as across-linkers. At a low pH, protonation of the phenolic group of tannic acid (TA) led to a high release of TA from CTZ, but at a higher pH, the release of TA from CTZ was decreased due to its lower protonation degree. The antibacterial activities of TA were further tested using a disk diffusion assay against *E. coli*. TA possesses antibacterial activities through multiple mechanisms, such as enzyme inhibition, enhanced penetrance of the membrane, and an impaired cytoplasmic membrane. As a result, the zone of inhibition (ZOI) of CTZ2 was 8 ​mm in comparison to the gentamicin (positive control), which was 9 ​mm; no ZOI was observed for the PBS (negative control). TA is released from the hydrogel in response to pH. In another study conducted by Villanueva and colleagues [141], an antimicrobial pH-responsive keratin/zinc oxide nanoparticles (nZnO) hydrogel was fabricated with tunable properties depending on the concentration of nZnO loaded in hydrogel and pH. Compared to basic pH (7–8), an acidic pH of 4 led to a reduced release of ZnO. Moreover, the antibacterial property of hydrogels loaded with nZnO (1%, 5%, and 10%) against *S. aureus* and *E. coli* was evaluated by Japanese industrial standards (JIS) and the disk diffusion method. Results from the JIS assay at a pH of 7.0 demonstrated a desirable antibacterial activity of nZnO (5%) and nZnO (10%) against both bacterial strains. Also, the disk diffusion assay results revealed that ZOI against *S. aureus* was 1.35 ​mm, and 1.90 ​mm, while against *E. coli,* it was 0.77 ​mm, and 0.96 ​mm for 5% and 10% concentrations, respectively. Sou and coworkers [142] synthesized a HA hydrogel loaded with an antimicrobial peptide [AMP, KK(SLKL)3 ​KK] as a cross-linker. Phase transition in their antibacterial hydrogel occurred above ​∼ ​pH 5.5–5.6. They also revealed that the AMP–HA-loaded hydrogel has no cytotoxicity with increased mechanical strength. The antibacterial property of their system was confirmed to kill both Gram-positive and negative strains. The AMP–HA-laden hydrogel also facilitated the infected wounds' healing procedure in an animal model. Khan and colleagues [143] produced a pH-sensitive blended hydrogel film as a wound dressing, which exhibited antibacterial properties. This novel hydrogel was developed by mixing PVA and Chitosan/Guar Gum (CS, GG) polymers. Also, Tetraethoxysilane (TEOS) was added as a cross-linker. The least swelling property of the hydrogels was observed around pH 7, whereas this behavior dramatically increased in acidic media (pH ​< ​7). This increased swelling rate was due to the action of NH^3+^, a cationic group in acidic pH, which is related to the degree of protonation. Consequently, these hydrogels were introduced as candidates for drug delivery systems with the power to control the drugs’ sustained release. The antibacterial activity of hydrogels with different concentrations of TEOS against Gram-negative *(P. aeruginosa)* and *(E. coli)* and Gram-positive *(S. aureus)* and *Bacillus cereus (B. cereus)* strains was examined through disk diffusion. The obtained results demonstrated that the interaction between the cationic nature of CS and lipophilic components of the bacterial membrane led to reduced bacterial growth. Moreover, rising amounts of TEOS can boost hydrophobic behavior and increase antibacterial activities against all strains. In another project reported by Sudarsan et al. [144], investigated the antimicrobial properties of a pH-sensitive silver nanocomposite hydrogel. These properties were evaluated by the well diffusion method with different concentrations of sodium ethylene glycol alginate (SEA) (9 ​Mm) and silver nanocomposite (SNC) (2.5 and 50 ​Mm) killed *(E. coli)* and *(S. aureus)*. Ciprofloxacin was used as the positive control. The results indicated that SEA-9/SNC-2.5 and SEA-9/SNC-50 hydrogels possessed more antibacterial activities against both strains than pure SEA hydrogel. It was concluded that this improved behavior is because of the interactions between Ag ​+ ​ions and bacterial cells in SEA-9/SNC-2.5 and SEA-9/SNC-50 hydrogels. Table 1 reviews the studies on pH-sensitive antibacterial hydrogels from 2010 to 2022.

**Table 1:** PH-sensitive antibacterial hydrogels as wound dressings.

Material(s)	Antibacterial agent(s)	Major finding(s)	Year, ref
Ag NPs Embedded in Smart Poly (*N*-Isopropylacrylamide)	Ag NPs	Significant antibacterial properties against *Acinetobacter* and *P. aeruginosa*	2011 [145]
Hyaluronan/PVA embedded with Ag NPs	Ag NPs	Has high antibacterial activity against *E. coli*	2013 [71]
Chitosan-PVA	Chitosan-PVA	Antibacterial effect against *Bacillus subtilis*, *S. aureus*, *P. aeruginosa*, and *E. coli* in pH 3 Antibacterial property against *Bacillus subtilis*, *S. aureus*, and *E. coli in* pH 7	2013 [146]
Synthesis of sodium alginate-based biodegradable pH-sensitive biopolymeric hydrogel	Sodium alginate- ethylene glycol- acrylic acid	Swelling properties at varied pH between acidic and alkaline Inherent Antibacterial activity against *E. coli* and *S. aureus in vitro* and *in vivo*	2016 [137]
pH-sensitive silane crosslinked injectable hydrogel for controlled release of NMS	NMS	The peak of swelling at the acidic phase, lowest at basic and at pH 7 Nontoxic behavior on HeLa cell lines Antibacterial activity against *E. coli*	2017 [147]
pH-Responsive 2-hydroxyethylmethacrylate/citraconic anhydride–modified collagen/ciprofloxacin	Ciprofloxacin	Antibacterial activity against *S. aureus*	2017 [148]
Multifunctional dressing (Gel-Derma) represents a colorimetric pH sensing array and drug-eluting	Gentamicin	Antibacterial activity against *P. aeruginosa* Soft mechanical properties Biocompatibility properties	2017 [149]
Carbon dots/chitosan	Carbon dot combination with chitosan	High mechanical properties Antibacterial activity against *S. aureus* Non-toxicity properties on L929 Improve wound healing	2017 [150]
Chitosan/PVA/silver sulfadiazine	Chitosan/silver sulfadiazine	Antibacterial activity against *E. coli*	2018 [14]
IA, AA/DEG	IA	Highly porous structure Antibacterial activity against *S. aureus* and *Bacillus cereus*	2018 [151]
Chitosan/PVP/silver sulfadiazine	Silver sulfadiazine	Antibacterial activity against *E. coli* High biocompatibility The least swelling at acidic pH and the highest swelling at neutral pH	2019 [10]
CaAlg/NPs/HAO	NPs/HAO	*In vivo* and *in vitro*, high antibacterial activity against *E. coli* and *S. aureus* Accelerate formation of blood vessel and healing of wound	2019 [152]
Oxidized starch/ZnO NPs	ZnO	Carboxylate anion leads to the highest swelling ratio at pH ​= ​7 Antibacterial properties against *S. aureus* and *E. coli*	2019 [153]
Silver(I)/Poly (2-hydroxyethyl acrylate/IA)	Silver/IA	Synergistic antibacterial effect of silver and IA against *MRSA* and *MMSA* Non-toxic on MRC-5 ​cells	2019 [154]
Cellulosic/chitosan	Chitosan	Swelling occurs at the low level of pH because the amino group of chitosan was protonated Antibacterial activity against *E. coli*, *S. aureus*, and *L. monocytogene*	2020 [155]
Chitosan with thyme oil cyclodextrin	Thyme oil and cyclodextrin	At pH ​= ​4 showed an increased swelling ratio and enhanced drug release Cell viability on L929 ​cells Antibacterial ability against *E. coli* and *S. aureus*	2020 [77]
CA cross-linked PVA/nano silver	Nano silver/CA	the pH of swelling depended on the amount of CA; drug release occurs at pH ​= ​7.4 Synergistic antibacterial effect of CA and nano silver against *E. coli* and *S. aureus*	2020 [78]
pH-responsive hydrogel-Ag NPs	AgNPs	Significant release at pH 7.4 or 10 Antibacterial activity against *P. aeruginosa* and *S. epidermidis in vitro* Cell viability on human foreskin fibroblast cell line (HFFs)	2021 [156]
pH-sensitive based dextran and peptide/ceftazidime hydrogel	peptide/ceftazidime	Can inhibit multidrug-resistant bacteria colonizing Promote wound healing Boost the adhesion of epithelial cells	2022 [157]
Self-healing/pH-responsive/polysaccharide/quaternised chitosan (QCS) -based hydrogel	QCS	Release drugs in acidic pH Antibacterial activity was increased by the photothermal-radiation method	2022 [158]
chitosan–graft-poly (hydroxyethyl methacrylate) (HEMA) hydrogel	(CHI-HEMA)	Antimicrobial ability against gram-negative bacteria and gram-positive bacteria	2022 [159]
PVA-bacterial cellulose-functionalized-Graphene oxide- Curcumin (PVA-BC-f-GO)	GO- Curcumin	Antibacterial activity against *S. aureus, E. coli,* and *P. aeruginosa* have physical-mechanical properties	2022 [160]

**Abbreviations:** (AA) Acrylic acid; (CA) Citric acid; (DEG) Diethylene glycol; (IA) Itaconic acid; (NMS) Neomycin sulfate; (PVA) Polyvinyl alcohol; (Ag NPs) Silver nanoparticles; (HFFs) Human foreskin fibroblast cell line; (ZnO NPs) Zinc oxide nanoparticles; (HAOs) hyaluronan oligosaccharides; (QCS) quaternised chitosan; (HEMA) hydroxyethyl methacrylate.

### 6.2 Thermo-sensitive antibacterial hydrogels as wound dressings

**Fig. 5:**
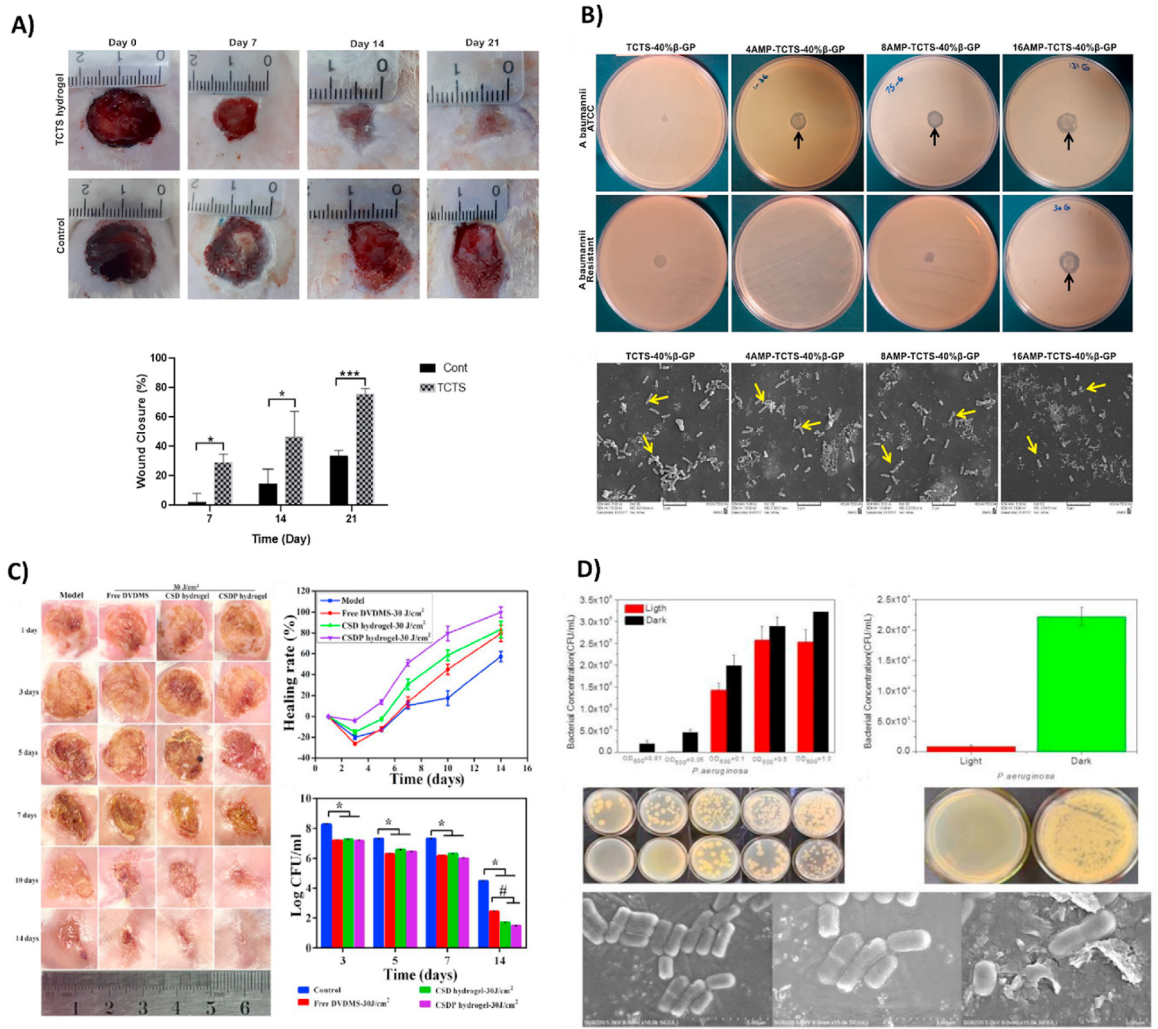
(A) Wound closure after treatment with TCTS hydrogel and gauze as control at days 7, 14, and 21. Reproduced with permission [169]. Copyright 2020, Elsevier. (B) Antibacterial behavior of TCTS -40%β -GP and AMP -TCTS -40%β -GP hydrogels against ATCC and resistant *A. baumannii*. All AMP -TCTS -40%β -GP hydrogels possess antibacterial activity against strains. SEM images of resistant *A. baumannii* are treated with TCTS -40%β -GP and AMP -TCTS - 40%β -GP hydrogels. Reproduced with permission [171]. Copyright 2020, Elsevier. (C) Evaluation of antibacterial activity in infected wound treated by CSDP hydrogel-PACT against MDR-*S. aureus* at days 1, 3, 5, 7, 10, and 14. Reproduced with permission [5]. Copyright 2020, American Chemical Society. (D) Antibacterial activity of the vesicles and prototype (20% TCDA vesicles contain photosensitive antibacterial agents) hydrogel against *P. aeruginosa*. SEM micrographs of *P. aeruginosa* bacteria grown on vesicles containing photosensitizer. (Right: *P. aeruginosa* with irradiated vesicles; Middle: *P. aeruginosa* with vesicles, in the dark; Left: *P. aeruginosa*). Reproduced with permission [197]. Copyright 2020, WILEY.

As already mentioned, some hydrogels are temperature-sensitive or thermo-responsive. Most of them are used for wound dressing and are also antibacterial. This antibacterial property could be due to metal nanoparticles or antibacterial drugs or the intrinsic property of polymers used in hydrogel structure. In recent decades, biomedical engineers have fabricated antibacterial thermo-responsive hydrogels and utilized them for different wound dressings. In this direction, *in vitro*, *in vivo*, and *ex vivo* experiments have shown that almost all improve wound healing (Table 2). For instance, Mi et al. [161] fabricated a thermo-sensitive multifunctional antimicrobial wound dressing hydrogel based on ABA triblock copolymers containing poly (*N*-isopropyl acrylamide) (PNIPAM) and [poly(N-1-(ethoxycarbonylmethyl)-*N*- (3-acryloylamino-propyl)-*N*,N-dimethyl ammonium salicylate)] (PCBAA-1-C2 SA) segments. It consists of a positive-charged hydrolyzable betaine ester loaded with an antimicrobial agent (inner B block) surrounded by two blocks of thermo-sensitive PNIPAM (outer A blocks). They synthesized it through reversible addition-fragmentation chain transfer (RAFT) polymerization. At body temperature, this copolymer solution is converted to a gel. *In vitro* studies have indicated that the use of this synthetic hydrogel with the controlled release of drugs and small molecules can diminish the risk of infection in wounds and enhance the healing process**.** Temperature-sensitive chitosan-agarose hydrogels were synthesized and characterized by Miguel et al. [162] have shown that this biocompatible hydrogel has antibacterial properties at doses greater than 188 ​μg/mL of chitosan. *In vivo* studies showed that the hydrogel could heal wounds without causing an undesirable inflammatory granulomatous reaction. Makvandi and colleagues [163] mixed Ag NPs, HA, and corn silk extract to fabricate an antibacterial thermo-sensitive hydrogel that enhanced wound closure and healing *in vitro*. A concentration of AgNPs≥1.7 ​μg/mL showed a 100% inhibitory effect on all tested microorganisms. Several smart hydrogels have been utilized for drug delivery systems and wound care. Also, Arafa and coworkers [164] assorted gold nanoparticles with Pluronic® F127 (PF127) and hydroxypropyl methylcellulose (HPMC) to fabricate a thermo-sensitive antibacterial hydrogel that can be used for burn wound healing and transdermal drug delivery. Xia et al. [64] developed an antibacterial chitin whisker (CW)/carboxymethyl chitosan nanoparticles (CMCS NPs)/thermo-responsive hydroxybutyl chitosan (HBC) composite hydrogel (CW/NPs/HBC-HG) for chronic wounds. They dissolved the wide-spectrum antibiotic (Linezolid) in the hydrogel before gelation and encapsulated recombinant human epidermal factor in the NPs. The *in vivo* study on chronic wounds in diabetic rats observed an acceleration in angiogenesis, collagen deposition, and enhanced re-epithelialization. Zhao et al. [165] fabricated a novel thermo-responsive antibacterial chitosan/β-glycerophosphate hydrogel loaded with β-cyclodextrin-curcumin. It also showed an anti-oxidative anti–NF–κB signaling capacity and improved cutaneous wound infection in rats. Another thermo-sensitive antibacterial hydrogel comprised of galactose modified xyloglucan (mXG) and hydroxybutyl chitosan (HBC) was developed by Zhang and colleagues [166], who demonstrated that this hydrogel not only exhibits good temperature sensitivity and effectively prevents recurrent adhesion after adhesion lysis but improves wound healing, reduces scarring, and prevents bacterial growth in the wound site in a rat model. The MIC value for *P. aeruginosa* is in the concentration range of HBC ≥500 ​μg/mL. This hydrogel inhibited the growth of Gram-negative strains such as *E. coli* and *P. aeruginosa* more than Gram-positive *S. aureus.* One of the important experiments in this section is on Methicillin-Resistant *S. aureus*-infected wound healing. Yan et al. [167] synthesized a smart sprayable *in situ* forming hydrogel that responds to skin temperature. It contains poly (*N*-isopropylacrylamide_166_-co-n-butyl acrylate_9_)-poly (ethylene glycol)-poly (*N*-isopropylacrylamide_166_-co-n-butyl acrylate_9_) copolymer (P(NIPAM_166_-co-nBA_9_)-PEG-P(NIPAM_166_-conBA_9_) (PEP) and Ag NPs-decorated reduced GO nanosheets (Ag@rGO, or AG). This PEP-AG hydrogel improved the healing of MRSA-infected wounds *in vitro* and *in vivo*. A NO-releasing thermo-sensitive hydrogel (GSNO-PL/AL) consisting of S-nitrosoglutathione (GSNO), pluronic F127 (PL), and alginate (AL) was designed and fabricated by Cao et al. [168] They showed wound repair in Gram-positive MRSA and Gram-negative multidrug-resistant *P. aeruginosa* (MRPA) infected wounds in the mouse burn wound model. In a study conducted by our group, a chitosan-based thermo-sensitive (TCTS) hydrogel was fabricated for non-healing extensively drug-resistant (XDR) clinical isolates of *A. baumanii* infected wounds [169]. Despite the intrinsic antibacterial property of chitosan, no antibacterial activity against investigated strains was observed from the results of disk diffusion assay. Surprisingly, a complete re-epithelialization on day 14 post-surgery in infected skin of rats was reported. The result of a colony count assay revealed that the TCTS hydrogel group depicted a remarkable reduction in the number as compared with the control (Fig. 5A).

**Table 2:** Thermo-sensitive antibacterial hydrogels as wound dressings.

Material(s)	Antibacterial agent(s)	Major finding(s)	Year, ref
*N*-isopropylacrylamide and Ag NPs	Ag NPs	Antibacterial against Gram-positive (*S. epidermidis*) and Gram-negative (*E. coli*) bacteria *in vitro* Thermo-responsive behavior	2014 [172]
Chitosan and Poly (N, *N*-diethylacrylamide) loaded gentamicin/ciprofloxacin	Chitosan and gentamicin or ciprofloxacin	Thermo-responsive hydrogel with proper adhesion of the dressing Drug loading capacity *In vitro* antibacterial activity against *S. aureus* and *P. aeruginosa*	2014 [173]
Chitosan/PNIPAAm modified cotton	Modified cotton fabrics	A drastic decrease in colonization of *E. coli* and *S. aureus* up to 99%	2016 [174]
Poly(*N*-isopropylacrylamide), poly(l-lactic acid-co-ϵ-caprolactone), and ciprofloxacin	Ciprofloxacin	Thermo-responsive swelling behavior Antibacterial activity killed *E. coli* and *S. aureus* Biocompatible and nontoxic on L929 Improve *in vivo* wound closure	2017 [175]
Poly(*N*-isopropylacrylamide), cellulose nanocrystals, and metronidazole	Metronidazole	Drug loaded potential Thermo-responsive hybrid gel Antimicrobial effect	2017 [176]
Poly (di (ethylene glycol) methyl ether methacrylate, poly (l-lactic acid-co-e-caprolactone), and ciprofloxacin	Ciprofloxacin	Thermo-responsive and drug-loaded capacity Antibacterial activity against *E. coli* and *S. aureus* *In vitro* safety on L929 ​cells and biocompatibility *In vivo* wound healing	2017 [177]
M-Arg, NIPAAm, *N*, *N′*-methylene bisacrylamide, CHX, and polyhexamethylene guanidine phosphate	CHX	Antibacterial thermo-sensitive hydrogel (killed *S. aureus* and *E. coli*) Non-toxicity *in vivo* and *in vitro* Anti-protein adsorption property Accelerated the full-thickness treatment of the wound	2018 [178]
Methylcellulose and silver oxide nanoparticles	Silver oxide nanoparticles	Injectable thermo-sensitive hydrogel About 99.9% of antibacterial activity *In vivo* burned skin treatment	2018 [179]
PNIPAm-alginate and AgNPs	AgNPs	Thermo-responsive antibacterial AADs i*n vivo* wound healing	2019 [180]
Thermo-responsive chitosan	Chitosan	Cell viability on Hu02 fibroblast cells Antibacterial activity against XDR *Acinetobacter baumannii in vitro* and *in vivo* Promote re-epithelialization	2020 [169]
Poloxamer 188 and poloxamer 407 and gentamicin	Gentamicin	Injectable hydrogel with antibacterial activity against *E. coli*, ​*B. cereus*, ​*S. aureus*, and MRSA	2021 [181]
Cotton fibers, cyclodextrins (HP-β-CD, DM-β-CD, and β-CDP), curcumin, (CS-*g*-PVCL) polymer, and citric acid	Cyclodextrins and curcumin	Non-toxic, antibacterial, and antioxidant properties	2021 [182]
Pluronic ​F-68 and Pluronic F-127, loaded with ​glucose oxidase (GOx@F68/F127)	glucose oxidase	100% antibacterial activity for ​*Staphylococcus aureus* ​at 0.65 ​μg/mL and 100% for ​*Escherichia coli* ​at 0.6 ​μg/mL High biocompatibility promote wound healing; (GOx@F68/F127) 98% VS control group 30%	2022 [183]
hydroxypropyl chitosan (HPCS) and poly(*N*-isopropylacrylamide) (PNIPAM) cross-linking by β-cyclodextrin (β-CD) and adamantyl (AD) and dipotassium glycyrrhizinate (DG); (HP-3/DG10)	dipotassium glycyrrhizinate (DG)	Antibacterial activity against S.*aureus* and anti-inflammatory properties Biocompatibility Improved tissue remolding and collagen deposition	2022 [184]
RA-Amps, RADA16 with (Amps), PNIPAM, MGF E peptide (PNI/RA-Amps/E)	antibacterial peptide (Amps)	Thermo-responsive, injectable and compatible with good mechanical properties Enhance collagen generation and accelerate epithelialization at the wound site	2022 [185]
vinyl carboxymethyl chitosan (CG) and graphene (GM) and *N*-isopropylacrylamide (NIPAM) and Ciprofloxacin Hydrochloride; (NIPAM-CG/GM)	Ciprofloxacin Hydrochloride	Thermo-responsive with drug release at a physiological temperature of 37 ​°C and antibacterial activity against *S. aureus* or *E. coli*	2022 [186]
Pluronic F127, PF127 and a complex of zinc and metformin (ZnMet); (ZnMet-PF127)	zinc and metformin (ZnMet)	The sparable, thermo-responsive hydrogel Acceleration in the healing of traumatic skin defect and burn skin injury by enhancing cell proliferation, angiogenesis, collagen formation Anti-bacterial activity against *S. aureus or E. coli* at the wound site	2022 [187]

**Abbreviations:** (AADs) Active adhesive dressings; (CHX) Chlorhexidine diacetate; (CW/NPs/HBC-HG) Chitin whisker and carboxymethyl/chitosan nanoparticles and hydroxybutyl chitosan; (GS) Gentamicin sulfate; (M-Arg) Methacrylate arginine; (NIPAAm) *N*-isopropyl acrylamide monomers; (XDR) Extensively drug-resistant.

An Amikacin encapsulated smart hydrogel was fabricated recently with antibacterial activity and suitable mechanical properties and accelerated the wound healing processes *in vivo*. These studies showed that wound closure was achieved entirely in 21 days in a rat model [170].

All of the mentioned studies involved some antibacterial experiments (*in vivo* or *in vitro*) on their synthetic thermo-sensitive hydrogels, and most of them confirmed an inhibitory growth effect against *E. coli* or *S. aureus* or both of them. In another study, a thermo-responsive chitosan hydrogel was fabricated and loaded with different concentrations of AMP [171]. Recent studies have demonstrated the antibacterial effects of AMPs, which are promising novel antibiotics for treating multi-drug-resistance bacteria. The antibacterial activity of AMPs varies widely, such as increasing bacterial membrane permeability, preventing intracellular activities, and leakage of cytoplasmic ingredients, which leads to bacterial cell death. Antibacterial properties of hydrogels containing different concentrations of AMPs (0, 4, 8, and 16 ​μg ​ml-^1^) against both standard strain and resistant *A. baumannii* bacteria were evaluated by disk diffusion method and SEM (Fig. 5B). Results from disk diffusion indicated ZOI against both bacterial strains and resistance for hydrogel with AMP (16 ​μg ​ml ^– 1^). Also, the observation from SEM proved that there is an inverse connection between the growing number of bacteria and AMP concentration. Thus, the bacteria growth was dramatically lowered when the concentration of AMP in the hydrogel was increased.

### 6.3 Radiation/photo-sensitive antibacterial hydrogels as wound dressings

Some synthetic antibacterial hydrogels for healing purposes are sensitive to radiation or light. This smart hydrogel shows antibacterial activity based on photo-thermal inactivation in bacteria and controllable drug/NPs release in the lesion [188]. What's more, radiation can trigger photo-biochemical reactions that inhibit infection and boost the healing process at the chronic wound site. Table 3 summarizes different antibacterial radiation-sensitive hydrogels synthesized for wound treatment between 2010 and 2020. Along these lines, Hong and Sun [26] fabricated a rose bengal (RB)/PVA hydrogel by the freeze-thawing process. This photo-induced hydrogel showed antibacterial behavior under UVA (365 ​nm) and fluorescence exposure against *E. coli* and *S. aureus*. However, the study was not performed on the wound site. Shi et al. [189] designed a photo-cleavable caged ciprofloxacin PEG hydrogel. Under UVA exposure (365 ​nm), 23.4 ​± ​5.2% of ciprofloxacin was released and limited the growth of *S. aureus in vitro*. It was considered a promising “spray-on” hydrogel for wound dressing applications. Similarly, Zhang and coworkers [190] combined a small amphiphilic peptide with a fullerene derivative to enhance mechanical properties. This hydrogel was exposed to white light (0.1 ​W cm-1) for 10 ​min and showed antibacterial activity against a multiantibiotic-resistant *S. aureus* strain. Another cross-linkable hydrogel sensitive to blue light was fabricated by Wang et al. [191] designed a double-network hydrogel with two different chitosan chains (catechol-modified methacryloyl chitosan, CMC; methacryloyl chitosan, MC). This light-sensitive hydrogel demonstrated nearly 100% antibacterial activity towards *E. coli* and *S. aureus* and showed favorable hemostatic performance and a boost in combating wound infection *in vivo* (Fig. 7D). For MDR burn infections, Mai et al. [5] developed a multifunctional intelligent hybrid hydrogel of carboxymethyl chitosan and sodium alginate (CSDP) that was used for photodynamic antibacterial chemotherapy (PACT). They confirmed about 100% bacterial elimination, reduced inflammation, and promoted tissue regeneration after light exposure *in vitro* and *in vivo*. Near-infrared (NIR) can induce photo-thermal inactivation in bacteria and also release drugs from some synthetic hydrogels. Tao et al. [13] also confirmed that a hydrogel formed by methacrylate-modified gelatin and N, *N*-bis(acryloyl) cystamine-chelated Cu nanoparticles (Gel-MA/BACA-Cu NPs) could promote wound closure and decrease bacterial growth *in vivo*. Photo-thermal-induced bactericidal activity to reduce *P. aeruginosa* biofilm and the suspension was shown via the use of a gold nanorod-loaded poloxamer 407 hydrogel system after NIR exposure *in vitro* by Al-Bakri and Mahmoud [192]. They concluded that this hydrogel nanosystem could be effective for treating common skin infections. One of the fascinating multifunctional composite hydrogels for use as a dressing and drug carrier was developed by Liang et al. [31] This gel is injectable and contains hyaluronic acid-graft-dopamine and reduced GO via a horseradish peroxidase (H2O2/HPR) system. In addition to having excellent biochemical and biomechanical properties, this hydrogel system exhibited strong antibacterial effects against *E. coli* and *S. aureus in vitro* and *in vivo*.

**Table 3:** Radiation/photo-sensitive antibacterial hydrogels as wound dressings.

Material(s)	Antibacterial agent(s)	Major finding(s)	Year, ref
SA, PEO, Pluronic F127, and lavender oil	Lavender oil	Nontoxic on HFF-1 ​cells Proper mechanical properties UVB-triggered hydrogel Antibacterial property against *S. aureus* Anti-inflammation behavior	2016 [199]
GelMA, MeTro, and antimicrobial peptide Tet213	Antimicrobial peptide Tet213	Antimicrobial activity against Gram (−) and (+) bacteria (MRSA and *E. coli*) Improve porosity, degradability, swellability, mechanical property, and adhesiveness Sprayable visible-light-responsive crosslinking hybrid hydrogel Biocompatibility, biodegradation, and nontoxic *in vivo* and *in vitro* (NIH-3T3 cells and rat subcutaneous skin)	2017 [200]
BTDA, HEMA, PEGD, chitosan, and methylene blue	Chitosan	Post-UV irradiation responsive (660 ​nm) Antibacterial activity against *E. coli* and *S. aureus in vitro* and *in vivo*	2018 [201]
Rose bengal/polypyrrole hybrid poly(vinyl alcohol) hydrogel and rhEGF	Rose Bengal	Antibacterial activity against *E. coli* and *S. aureus in vitro* and *in vivo* at mild temperatures (45 ​°C) under dual light irradiation (808 ​nm NIR light and 550 ​nm VL irradiation)	2021 [202]
Graphene hybrid supramolecular hydrogel	Photothermally responsive active Graphene	Antibacterial behavior against MRSA Promote granulation-tissue formation by reducing inflammation, enhancing angiogenesis and collagen deposition in an acute wound infection model	2021 [203]
Ag2S quantum dot/mSiO2 NPs and 3-(trimethoxylmethosilyl) propyl methacrylate	Ag+	NP hydrogel with photothermal and photodynamic characteristics under 808 ​nm (NIR) light irradiation, with a photothermal conversion efficiency of 57.3% for releasing the Ag+ Antibacterial activity E. coli & MRSA (inhibition rate of 99.7% and 99.8%, respectively) Enhancing collagen coverage area and bacterial clearance *in vivo*	2022 [204]
Ag nanoparticles/phosphotungstic acid-polydopamine nano-flowers and chitosan (CS)/gelatin (GE)	Ag+	Anti-bacterial activity against both gram-negative *E. coli* and gram-positive *S. aureus* with releasing Ag ​+ ​under NIR and accelerating wound healing *in vivo*	2022 [205]

**Abbreviations:** (BTDA) 3,30,4,40-benzophenone tetracarboxylic dianhydride; (GelMA) Gelatin methacryloyl; (HEMA) 2-hydroxyethyl methacrylate; (MeTro) Methacryloyl tropoelastin; (PEGD) Poly ethylene glycol diacrylate; (GT-DA/chitosan/CNT) gelatin-grafted-dopamine and polydopamine-coated carbon nanotubes; (PDA/Cu-CS) polydopamine (PDA) and copper-doped calcium silicate ceramic (Cu-CS).

In summary, all of these radiation-sensitive composite hydrogels that can be used for wound dressing were biocompatible and showed excellent antibacterial activity even against some MDR bacteria *in vitro*, *in vivo*, or both [193].

**Fig. 6:**
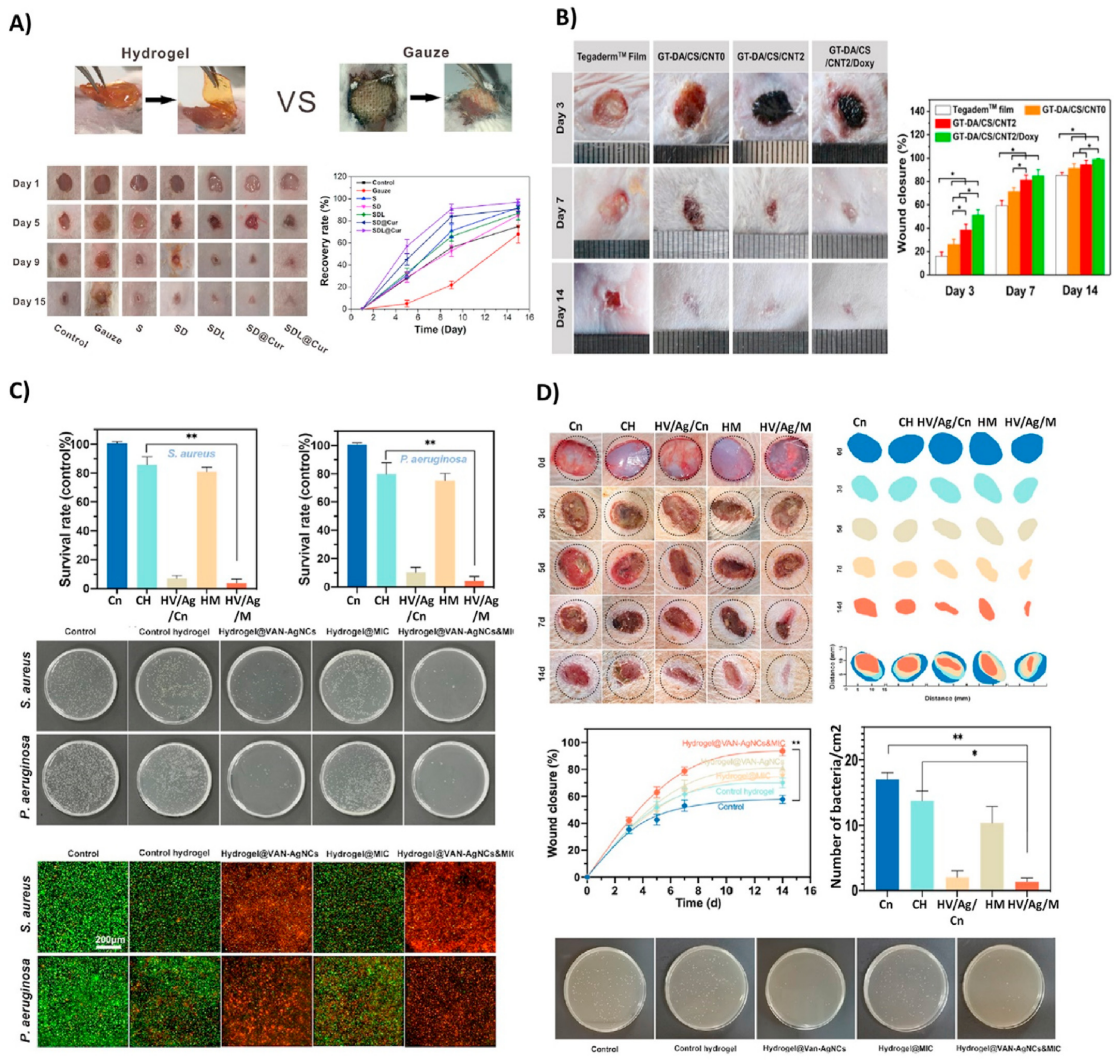
(A) Representative imaging of *S. aureus*-infected wound in a mouse model (15-day wound healing treated with the SDL@Cur hydrogel SDL, SD, S, and gauze). Reproduced with permission [198]. Copyright 2021, American Chemical Society. (B) Wound infection treated with TegadermTM film, GT-DA/CS/CNT0, GT DA/CS/CNT2, and GT-DA/CS/CNT2/Doxy hydrogel and the percentage of wound closure for each group. Reproduced with permission [195]. Copyright 2019, Elsevier. (C) Antimicrobial activity treated with hydrogel VAN-AgNCs and hydrogel MIC@NIM, hydrogel VAN-AgNCs-MIC@NIM, and control against *S. aureus* (A) and *P. aeruginosa*. (D) Infected diabetic wound healing treated with hydrogel VAN-AgNCs, hydrogel MIC@NIM, hydrogel VAN-AgNCs-MIC@NIM, and the control. Also, the wound closure and colony count results after seven days are presented. Reproduced with permission [217]. Copyright 2021, American Chemical Society. (Cn: Control, CH: Control Hydrogel, HV/Ag/Cn: hydrogel VAN-AgNCs, HM: hydrogel MIC@NIM, HV/Ag/M: hydrogel VAN-AgNCs-MIC@NIM).

A multi-functional nanocomposite dressing made from tungsten disulfide nanosheets, dodecyl-modified chitosan (FCS), dialdehyde-functionalized PEG (PEG-CHO), and ciprofloxacin with high sensitivity to NIR irradiation showed a solid bactericidal effect both *in vitro* and, in a mouse infected wound model. The construct also revealed an anti-oxidation property, which caused reduced inflammation and subsequently promoted the wound-healing processes. Recently, Ma and coworkers [194], showed the *in vitro* and *in vivo* antibacterial activity of a hydroxypropyl chitin (HPCH), tannic acid (TA), and ferric ions (Fe^3+^) composite hydrogel under 10 ​min of NIR laser irradiation. This smart injectable hydrogel is cytocompatible and promotes wound healing. Another composite hydrogel consisting of gelatin-grafted-dopamine/chitosan/carbon nanotubes (GT-DA/chitosan/CNT) and doxycycline with photothermal antibacterial activity fabricated by Liang et al. [195] demonstrated an antioxidant, adhesive, and conductive characteristics. This study revealed that the GT-DA/chitosan/CNT under NIR irradiation could eradicate infection and boost healing of the full-thickness infected wound *in vivo* (Fig. 6B). The hot ions effect of Cu through the photothermal activity of a polydopamine/Cu/chitosan (PDA/Cu-CS) composite hydrogel greatly impacted antibiotic-resistant infection wound healing [196]. This synthesized hydrogel improved cell proliferation and angiogenesis *in vitro* and *in vivo* in methicillin-resistant *S. aureus* and *E. coli* wound infection models. Prospects for these antibacterial smart hydrogels could be the mixture of photodynamic and photothermal therapy to achieve more efficient strategies for wound treatment methods (Fig. 8A and B). In another study by Mai and coworkers, a hydrogel-based nano-delivery system was developed with antimicrobial and skin re-epithelialization properties. They was considered for photodynamic antimicrobial chemotherapy (PACT) in the treatment of burn wound and could be a biocompatible delivery system of various therapeutics and nanoparticles. These CSDP nanohybrids also displayed an effective inhibition against MDR *S. aureus* biofilm formation (Fig. 5C) [5]. A further smart photosensitive antibacterial hydrogel was fabricated by Zhou et al. [197] They designed a hydrogel containing tryptophan-modified trithiophene aldehyde (3T-CHO) (3 ​TT) as a photosensitizer to create a dressing with detection and antimicrobial applications. Experiments showed that the released 3 ​TT (photosensitizer and antibacterial agent) was associated with toxins (pyocyanin released by *P. aeruginosa* or hemolysin released by *S. aureus*). Toxin lysed the vesicles, which led to the release of fluorescein for the detection of infections and displayed bactericidal effects on *P. aeruginosa* after light irradiation (Fig. 5D).

**Fig. 7:**
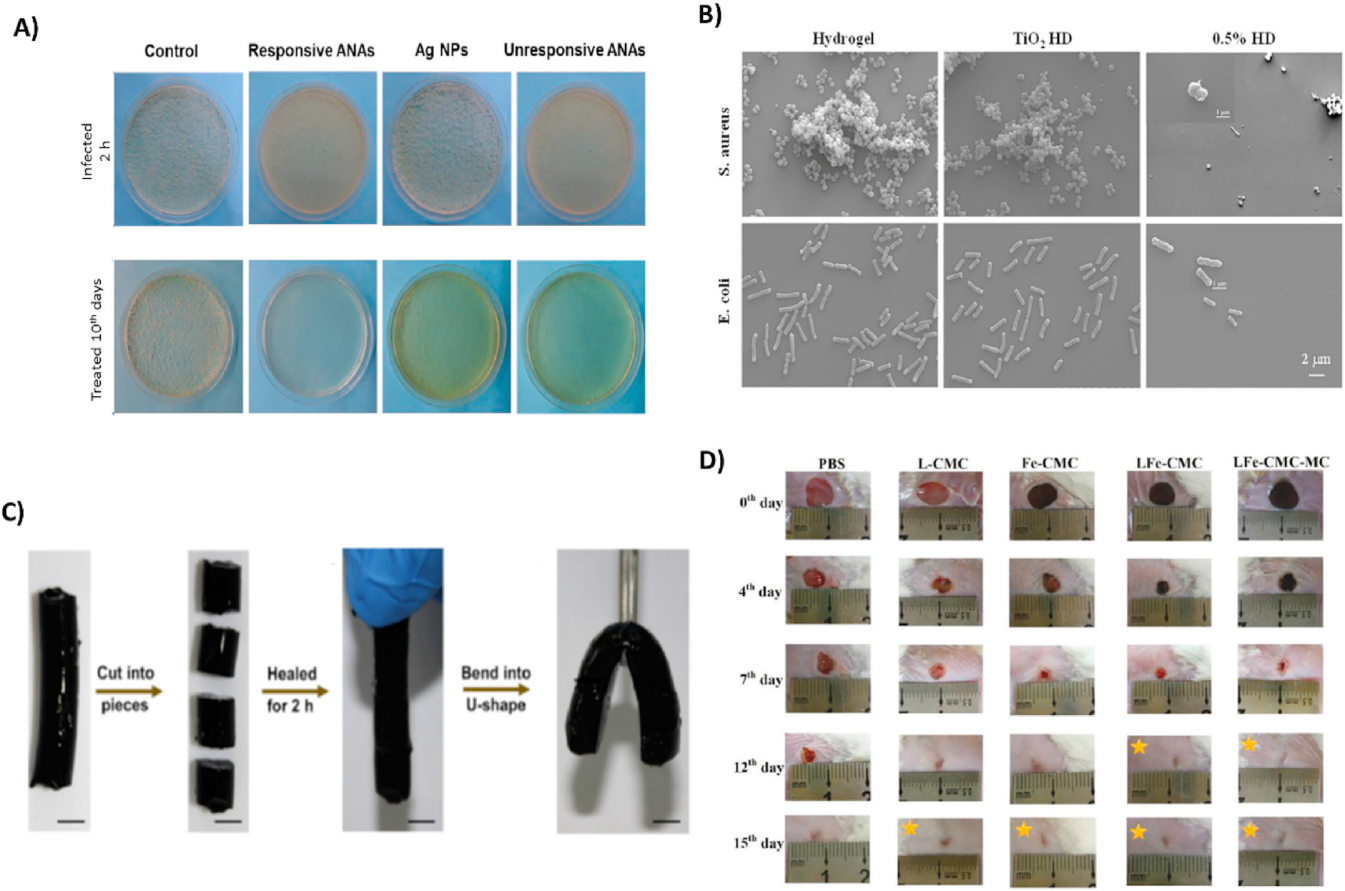
(A) Cultured MRSA-infected wounds of a rat wound healing model after ten days. Reproduced with permission [239]. Copyright 2020, American Chemical Society. (B) SEM micrographs of *E. coli* and *S. aureus* bacteria are grown on TiO2 hydrogel. An increased TiO2 concentration resulted in a significant reduction in the number of bacteria on hydrogels. Reproduced with permission [216]. Copyright 2017, Elsevier. (C) A hydrogel was cut into four pieces and reassembled after 2 ​h incubation at 25 ​°C. Reproduced with permission [1]. Copyright 2019, Elsevier. (D) Macroscopic images of *S. aureus*-infected rat skin after 15 days of treatment with PBS solution (as the control group), L-CMC, Fe-CMC, LFe-CMC, and LFe-CMC-MC hydrogels. Reproduced with permission [240]. Copyright 2019, WILEY.

**Fig. 8:**
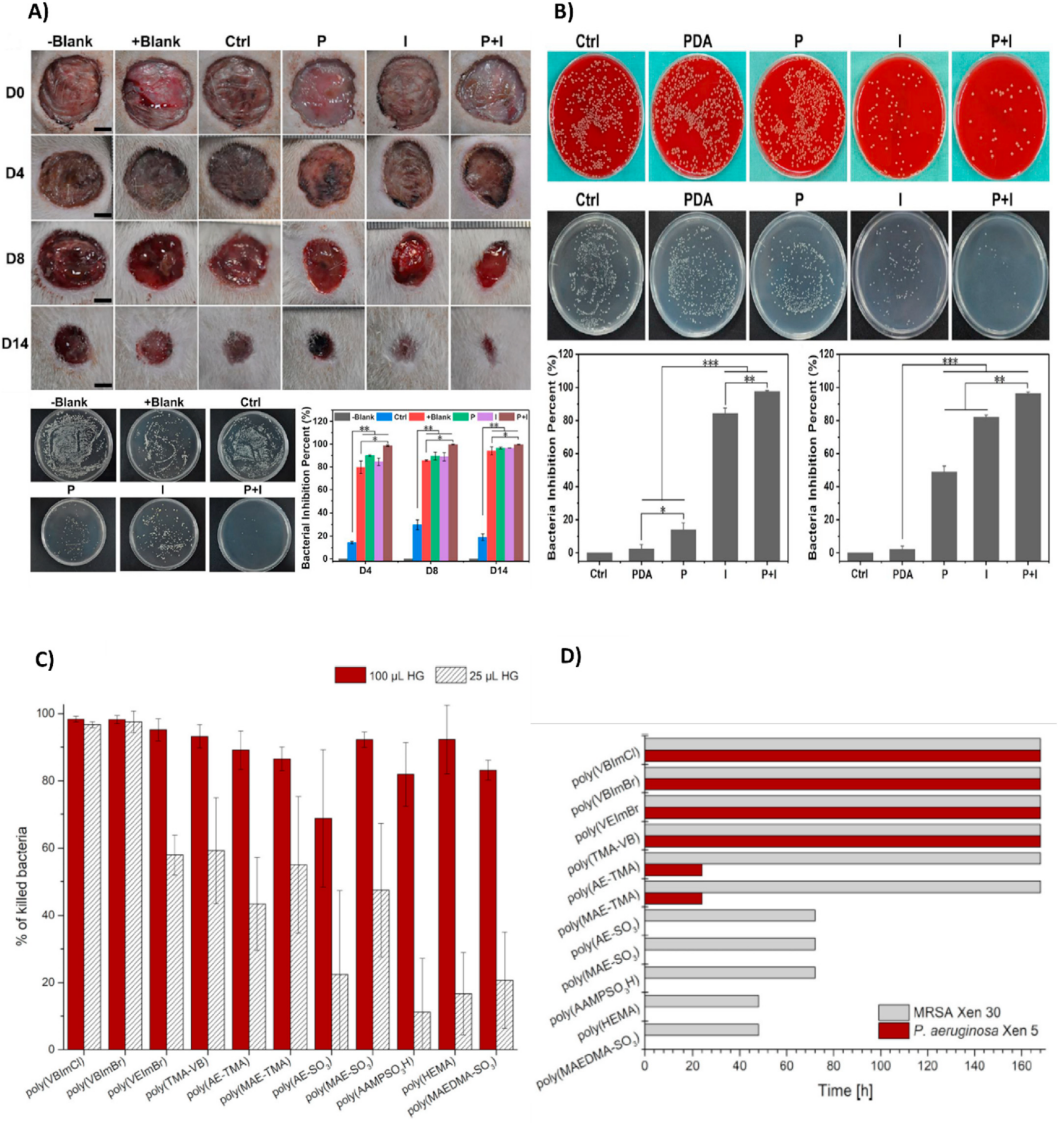
(A) Investigation of antimicrobial properties and wound healing percentage of hydrogels at different days 4,8 and 14, *in vivo*. Colony count assay against wound infection with *E. coli*, *in vivo*. (B) Images from the *in vitro* antibacterial activity of the hydrogels groups of Ctrl, PDA, P (PDA hydrogel with laser irradiation), I (PDA/Cu-CS hydrogel), and P ​+ ​I (PDA/Cu-CS hydrogel with laser irradiation) against *E. coli* and MRSA. Reproduced with permission [196]. Copyright 2020, American Chemical Society. (C) Antibacterial effect's period of the hydrogels against *P. aeruginosa* and MRSA. (D) Antibacterial effect of hydrogels against MRSA. Reproduced with permission [288]. Copyright 2020, WILEY.

A multifunctional antifouling and antimicrobial zwitterionic sulfobetaine acrylamide hydrogel with laponite-dopamine (LAP-DMA) & curcumin was also fabricated by *Dai* et al. [198] The sustained release of curcumin happened under UV-irradiation. The SDL@Cur hydrogel, composed of SD (prepared by SBMA, MBA, and DMA monomers) with LAP and curcumin, was able to heal the infected wound with complete re-epithelialization and development of new connective tissue on day 15, *in vivo* (Fig. 6A)*.*

### 6.4 Biological smart antibacterial hydrogels as wound dressings

#### 6.4.1 Enzyme-responsive antibacterial hydrogels

Enzymes display a critical role in many biological processes in tissue engineering and drug delivery. Enzymes act as a signal for the drug delivery system when they are placed at a specific site in the human body. In tissue engineering, MMPs are a group of enzymes that degrade the ECM proteins [206]. In recent years, enzyme-sensitive hydrogels have been developed. Specific hydrolytic enzymes, like glycosidases, lipases, proteases, and oxidoreductases like peroxidases, have been utilized for several schemes. Enzyme-sensitive hydrogels show various advantages such as specificity, regioselectivity and stereoselectivity [207]. Zuo et al. [208] synthesized an enzyme-responsive antibacterial Ag NP hydrogel, and its antibacterial effects against *S. aureus* were evaluated *in vitro* and *in vivo*. Methacrylate-WELQK-methacrylate (M-WELQK-M) was synthesized as a cross-linker. Silver can inhibit the replication of DNA and RNA and destroy the cell membrane. In this study, silver was subjected to the serine protease-like protein B (SplB) secreted by *S. aureus*. The researchers used three models, i.e., responsive Ag NP assemblies (ANAs), unresponsive ANAs, and Ag NPs, *in vitro*. The result showed that responsive ANAs (4 ​μg ​× ​mL−1) had increased antibacterial activity against MRSA. The reduced rates of responsive ANAs, unresponsive ANAs, and Ag NPs were 86.7%, 74.1%, and 64.6%, respectively. Many studies have revealed that a smaller Ag NPs increases antibacterial activity. In this study, however, the size of responsive ANAs was more significant than that of Ag NPs, while they revealed stronger antimicrobial effects. The researchers concluded that enzyme responsiveness is somehow directly related to antibacterial properties. The colony count experiment was performed to investigate the antibacterial activity of the samples against MRSA *in vivo*. As a result, responsive ANAs indicated the highest antibacterial effects and reduced bacterial colonies; see (Fig. 7A).

#### 6.4.2 ROS-responsive antibacterial hydrogels

Reactive Oxygen Species (ROS) play essential roles in metabolic processes, such as adjusting cell signaling, modulating inflammation, removing infections, and regulating protein functions [209]. Moderate ROS levels can affect physiological activities like promoting wound healing and eliminating bacterial invasions [210]. H_2_O_2_, HO^−^, O_2_^−^, and hypochlorous acid (HOCL) are critical ROS compounds [211,212]. The moderate values of H_2_O_2_ in the human body can activate cell signaling mediators such as vascular endothelial growth factor (VEGF), keratinocyte growth factor (KGF), and EGF [209,213]. Over-production of ROS may lead to incomplete wound healing, inflammation, scarring, and necrosis [214]. Infection throughout the inflammatory phase of wound healing is controlled with increased ROS levels [215]. Wang and coworkers [216], designed a PVA hydrogel incorporated with Ag/TiO_2_ by light-induced ROS and evaluated its antibacterial activity against Gram-negative and positive strains *in vitro* and *in vivo*. They used samples with different contents of Ag under two different ranges of visible light (VL). The results exhibited that the 0.5% Ag/TiO_2_ hydrogel could kill both *S. aureus* and *E. coli* strains in both ranges. However, the antibacterial activity of this hybrid hydrogel was significantly enhanced under 660 ​nm irradiation of VL. The morphology of both strains was observed under SEM (Fig. 7B). The results showed a decline in the number of bacteria in the sample treated with 0.5% Ag/TiO_2_ hybrid hydrogel. This may be due to the oxidized lipid membrane of bacteria under the ROS condition, which in turn can disrupt the bacterial membrane. As already mentioned, the photodynamic character of Ag/TiO_2_ hydrogel was evaluated under VL and 660 ​nm VL irradiation. The results showed that the amount of singlet oxygen (^1^O_2_) produced was directly related to Ag content under 660 ​nm laser radiation.

Furthermore, the hybrid hydrogel produced high local concentrations of hydroxyl radicals (^.^ OH) under VL and 660 ​nm radiation. In contrast to produced ^1^O_2_, the amount of produced OH under VL (not 660 ​nm) was affected with the concentration of AgNPs in the hydrogel. In conclusion, Ag/TiO_2_ hydrogel showed photocatalytic activity under VL and 660 ​nm irradiation. Results taken from *in vivo* experiments also revealed an effective antibacterial activity of 0.5% Ag/TiO_2_ hydrogel while promoting wound healing. In a study reported by Wang et al. [217] a hydrogel that responded to two stimuli (pH and ROS) was fabricated by bilayers of phenylboronic acid (BA) and gelatin. The combination of amide bond −NH2 in Gel and –COOH in 3-carboxy-BA and cross-linking with poly (vinyl alcohol) promoted pH- and ROS-sensitivity. Ultrasmall Ag nanoclusters (AgNCs) and vancomycin (VAN)-AgNCs as antibacterial agents were integrated into the hydrogel. After 48 ​h, only 70% of AgNCs and VAN were released at pH 7.4. Meanwhile, at pH 5.5, The release rate of AgNCs and VAN was enhanced and reached the 90% peak at 48 ​h. In addition, high ROS conditions caused a rapid release of VAN-AgNCs. However, sustained release of nimesulide (NIM) was observed under acidic phases. Antibacterial properties of the hydrogel against *S. aureus* and *P. aeruginosa* were evaluated in an animal model. *In vitro*, SYTO 9 and propidium iodide (PI) were utilized to estimate bacterial cell death. The live/dead pathogens ratio was observed by optical density (OD) at 600 ​nm (OD600). As the result, by rising the concentration of VAN-AgNC the antibacterial property was increased. To specify, 4 main groups including hydrogel@VAN-AgNCs, hydrogel@ VAN-AgNCs&MIC, hydrogel@MIC, and control hydrogel were examined (Fig. 6C). The result indicated that hydrogel@VAN-AgNCs&MIC and hydrogel@VAN-AgNCs showed a strong antibacterial effect against *S. aureus*, which peaked at 90 or 96%. For strains treated with control hydrogel and hydrogel@MIC, the bactericidal activity plunged to roughly 15%. A similar result was observed against *P. aeruginosa*. Furthermore, the antibacterial activity of hydrogels was assessed by colony count *in vivo* (Fig. 6D). The result revealed the higher bactericidal effect of hydrogel@VAN-AgNCs and hydrogel@VAN-AgNCs&MIC-treated wounds compared to the control group. Table 4 summarizes the studies on biological-sensitive antibacterial hydrogels from 2010 to 2022.

**Table 4:** Biological smart antibacterial hydrogels as wound dressings.

Biomaterials	Hydrogel types	Antibacterial agents	Major findings	Year, ref
Chitosan/MUG/(PNPG) as covalent modification	Enzyme-sensitive	Chitosan	Enzyme β-GUS used as a probe for the detection of *E. coli* Antibacterial activity against *E. coli*	2015 [218]
EPL	Enzyme-sensitive	EPL	HRP/H2O2 enzymatic crosslinking affected mechanical strength and degradation Non-toxic on L929 ​cell EPL possess an antibacterial effect against *S. aureus* and *E. coli* *In vivo*, EPL with inherent antibacterial activity prevent infection	2016 [17]
PCA	ROS-sensitive	AgNPs	AgNPs could affect the generation of ROS Excellent antibacterial properties against *P. aeruginosa* and *S. aureus* *In vivo*, enhance wound healing	2019 [219]
CeONs/AMP	ROS- sensitive	AMP	CeONs exhibited favorable ROS-scavenging abilities Biocompatibility on HaCaT cells Antibacterial activity *In vivo*, promote wound healing	2021 [220]
Arg-PEUU/CS-GMA	Enzyme-sensitive	Amino group in CSGMA and the guanidine pendant group in Arg-PEUU	Non-toxic on NIH-3T3 fibroblast cells and human vascular endothelial cells Have remarkable bactericidal effect against *E. coli* and *S. aureus*	2021 [221]
Covalent organic framework (COF) coloaded with silver nanoparticles (AgNPs) and ebselen (EBS) (Ag-TACON@EBS@PEG)	Enzyme-sensitive	EBS/Ag+	Bactericidal properties against Gram-positive and Gram-negative strains Have biocompatibility and anti-inflammatory property Enhance wound healing	2022 [222]
Sodium alginate and sodium hyaluronate- Doxycycline hydrochloride	ROS- sensitive	Doxycycline hydrochloride	It has dynamic mechanical properties Show antibacterial properties *in vitro*	2022 [223]
hydrogel-loaded hyper-branched poly-l-lysine (HBPL)	ROS- sensitive	(HBPL)	It has an excellent anti-oxidative effect Can inhibit the QS system of MRSA Develop wound healing and reduce infection *in vivo*	2022 [224]

**Abbreviations:** (AgNPs) Silver nanoparticles; (AMP) Antimicrobial peptide; (COF) covalent organic framework; (EBS) ebselen; (EPL) Epsilon-poly-l-lysine; (HBPL) hyper-branched poly-l-lysine; (MUG) 4-methyllumbelliferyl ẞ-D- glucuronide; (PCA) Polyvinyl alcohol/chitosan/silver nanoparticle; (PNPG) 4-nitrophenyl ẞ-D- glucuronide; (CeONs) Cerium oxide nanoparticles; (Arg-PEUU/CS-GMA) arginine-based poly(ester urea urethane) (Arg-PEUU) glycidyl methacrylate-modified chitosan.

### 6.5 Electro-sensitive antibacterial hydrogels as wound dressings

Recent studies have shown that electro-responsive hydrogels can be applied as dressings to control the wound-healing process [225]. This is intimately linked to the fact that the swelling behavior of electro-responsive hydrogels varies depending on the applied electric field; something which depends on various mechanisms like electro-osmotic, dynamic enrichment/depletion, electro-chemical, and Coulomb blockade phenomena. Their response can also be controlled by variables like ionic strength, pH, the composition of hydrogel, the presence of other chemical agents, swelling degree, etc. [226] However, some design limitations here include low elasticity, weak mechanical strength, and low stability [227,228]. Examples of such intelligent hydrogels include a recent study by Di Luca and colleagues [225], synthesized an electro-responsive graphene oxide hydrogel loaded with curcumin (Cur). They suggested a considerable development in the design of hydrogel by using GO to boost the electro-tunability of the releasing abilities as a consequence of both the high theoretical specific surface area and large drug loading capacity. They employed two compounds, such as gelatin (Gel) and trypsin (Try), with various biological capabilities. The gel is given a great deal of attention as one of the best candidates for wound dressing due to its biocompatibility and non-immunogenicity, ability to promote hemostasis and protect epidermal growth factor from proteolysis. In contrast, try is used to remove necrotic tissues by proteolysis. AAm and PEGDMA were used as crosslinkers. This hydrogel was assessed at different voltages (0–48 ​V), and the biological molecule's highest and lowest release rates were observed at 0 and 24 ​V, respectively. The antibacterial effect of Cur against MRSA was subsequently determined. The result showed that hydrogels with a high concentration of Cur (200–500 ​μg ​mL^−1^) could reduce 6 logs of colony forming unit (CFU).mL^−1^ [225]. Table 5 summarizes studies conducted on electro-sensitive antibacterial hydrogels between 2010 and 2022.

**Table 5:** Electro-sensitive antibacterial hydrogels as wound dressings.

Biomaterials	Antibacterial agents	Important findings	Year, ref
QCS-polyaniline/OD	QCS/polyaniline	Electrical stimulation enhances the proliferation of C2C12 myoblast Antibacterial effect against *E. coli* and *S. aureus* Antibacterial activity against *E. coli in vivo* and improve wound healing	2015 [229]
Starch/graphene oxide	Graphene oxide	High rheological activity Antibacterial activity against *E. coli* and *S. aureus*	2018 [230]
PDA@ AgNPs/CPHs	AgNPs	Mechanical and electrochemical properties Antibacterial activity killed *E. coli* and *S. aureus* *In vivo*, control infection and promote healing	2019 [231]
zwitterion poly[3-(dimethyl(4-vinylbenzyl) ammonium) propyl sulfonate] (SVBA)/poly-acrylamide	poly (AAm-co-SVBA) (PAS)	Have significant mechanical and electroactive properties It has an excellent rheological property Have an antibacterial ability against *E. coli* and *S. aureus*	2022 [232]

**Abbreviations:** (QCS-polyaniline) Quaternized Chitosan-graft-Polyaniline; (OD) Oxidized dextran; (AgNPs) Silver nanoparticles; (PDA@ AgNPs/CPHs) poly dopamine decorated silver nanoparticles/conductive polymer-based hydrogels; (SVBA) zwitterion poly[3-(dimethyl(4-vinylbenzyl) ammonium) propyl sulfonate; (PAS) poly (AAm-co-SVBA).

### 6.6 Self-healing smart antibacterial hydrogels as wound dressings

Self-healing hydrogels possess the ability to heal automatically. This property can, in turn, increase hydrogel lifetime and thereby increase mechanical toughness. Dynamic covalent or non-covalent bonds are the basis of these smart hydrogels [233]. Combining these active interactions with stiffness is a significant challenge in designing self-healing hydrogels, as high stiffness requires solid crosslinks [234]. Self-healing hydrogels have gained rising consideration recently, especially in drug delivery, tissue engineering, and wound healing approaches [235]. As a wound dressing, these hydrogels can fill the irregularly shaped wound surfaces and, thus, provide a bed to ameliorate the natural procedure of wound healing [236]. Zhao et al. [1] designed and developed a self-healing hydrogel based on chitosan-g-polyaniline (QCSP) and benzaldehyde poly (ethylene glycol)-co-poly (glycerol sebacate) (PEGS-FA); see (Fig. 7C). The hydrogel was prepared by dynamic covalent bonds between amine groups from QCSP and benzaldehyde from PEGS-FA. The rapid and effective self-healing potency stemmed from a dynamic covalent Schiff-base network. The antibacterial activity of samples with different cross-linker concentrations (QCSP3/PEGS-FA0.5, QCSP3/PEGS-FA1.0, QCSP3/PEGS-FA1.5, and QCSP3/PEGS-FA2.0) was determined against *E. coli* and *S. aureus*. According to the results, QCSP3/PEGS-FA0.5 demonstrated the lowest antibacterial activity – 24% kill for *E. coli* and 91% for *S. aureus*.

Meanwhile, other samples displayed antibacterial activity over 99% and 100% for *E. coli* and *S. aureus*, respectively. The synergetic antibacterial effects of QCSP and PEGS-FA were the main reason for this fascinating result. A hydrogel based on ε-poly (l-lysine)-modified poly (vinyl alcohol) (CPVA-g-EPL)/chitosan (CS)/Ag complex was synthesized and then cross-linked with oxidized dextran. The dynamic Schiff base linkages led to enhanced self-healing ability. Ag nanoparticles (AgNPs) loaded in the hydrogel indicated antibacterial effects against *E. coli* and *S. aureus in vitro*. This hydrogel also showed accelerated wound healing in a mouse skin model*.* [237] Recently, Tavakolizadeh et al. [238] designed a self-healing double-network (DN) hydrogel based on oxidized salep/poly (vinyl alcohol). The self-healing capability of the hydrogel was observed by the interaction between the free aldehyde and amine components inside of the oxidized salep and ethylene diamine-modified salep (SaHEA) chain (OSEA) network as a cross-linker. Colony count and disk diffusion methods were performed to investigate the antimicrobial effects of PVA/OSEA DN hydrogels against *E. coli* and *S. aureus*. As a result, the number of colonies was less than that of colonies in control (the hydrogel without AgNP). In this study, the result of disk diffusion indicated that the inhibition zones of hydrogel PVA/OSEA DN with AgNP and Arnebia extract as antibacterial agents against both strains were approximately the same as that of a control group (Ciprofloxacin discs). Table 6 summarizes recent studies on self-healing smart antibacterial hydrogels from 2010 to 2022.

**Table 6:** Self-healing smart antibacterial hydrogels as wound dressings.

Material(s)	Hydrogel types	Antibacterial agent(s)	Major finding(s)	Year, ref
QCSP/benzaldehyde group functionalized PEGS FA	Self-healing	QCSP/PEGS-FA	Self-healing properties due to dynamic covalent between benzaldehyde groups from PEGS-FA and amine groups from QCSP High antibacterial activity against *E. coli* and *S. aureus* Enhance ECM synthesis, collagen deposition, and granulation tissue thickness High expression main growth factor VEGF, EGF, and TGF-b	2017 [1]
QCS/PEO99-b-PPO65-b-PEO99, PF127	Self-healing	QCS	Self-healing properties due to dynamic covalent Schiff-base bonds between amine groups (QCS) and benzaldehyde (PF127–CHO) Inherent bactericidal property against *E. coli* and *S. aureus* Promote wound healing	2018 [241]
Hydrogel based on QC/OD	Self-healing	QC/OD	Excellent self-healing because of reversible hydrogen and imine bonds Antibacterial activity against *E. coli* Non-toxic on NIH-3T3	2018 [233]
Injectable gelatin hydrogels/polyethene glycol dibenzaldehyde/Clindamycin	Self-healing	Clindamycin	Self-healing due to imine reaction between gelatin and diBA-PEG's chains Excellent cell viability Antibacterial activity against *S. aureus*	2018 [242]
Anti-bacterial hydrogel based on polyvinylpyrrolidone, acrylamide/1-vinyl-3-butylimidazolium/polyethylene glycol	Self-healing	VBIMBr	Anti-bacterial efficacy against *E. coli and S. aureus* Strong adhesive property Non-toxic on L929 ​cell Promote wound healing	2019 [243]
bFGF@PLGA/CHA	Self-healing	CHA	Dynamic imine and acyl hydrazone bond leads to self-healing abilities *In vitro* and *in vivo*, CHA has good antibacterial activity against *S. aureus and E. coli* *In vivo*, due to sustained release of bFGF, promotes wound healing	2019 [244]
Chitosan/oxidized konjac glucomannan/AgNPs	Self-healing	AgNPs	Biocompatibility on L929 *In vitro* antibacterial effect against *E. coli* and *S. aureus* *In vivo* antibacterial properties against *S. aureus* and promote wound healing	2020 [245]
QP-(DMAPMA)	Self-healing	QP4	Biocompatibility on CT-26 Antibacterial activity against *E. coli*	2020 [246]
CS/DABC	Self-healing	CS-DABC	Self-healing due to dynamic covalent between –NH2 and the –CHO on the surface of the CS-DABC Excellent antibacterial activity against *E. coli* and *S. aureus* High biocompatibility on L929	2020 [247]
N, O-CMC/OCS	Self- healing	N, O-CMC/OCS	Biocompatibility on NIH/3T3 cells Antibacterial properties against *E. coli* and *S. aureus*, *In vitro* Hemostatic properties	2021 [248]
Zwitterionic dextran-based hydrogels/CB-Dex and SB-Dex	Self- healing	CB-Dex and SB-Dex	Excellent cytocompatibility with NIH3T3 and L929 ​cells Antibacterial adhesion against *S. aureus* and *E. coli* Enhance wound treatment and pain relief *in vivo*	2021 [249]
Acrylic acid/1-vinyl-3-butylimidazolium/COOH-modified gum Arabic/aluminium chloride	Self-healing	aluminium chloride	Significant self-healing property due to interaction between -vinyl-3-butylimidazolium as an ionic liquid and covalent bonds High antibacterial activity against *E. coli, S. aureus*, and *C. albicans, In vitro* Promote wound healing, *In vivo*	2021 [250]
OCMC-DA, PVA, and CNF	Self-healing	(NEO)	Excellent self-healing ability and stretchability Anti-bacterial property against *E. coli* and *S. aureus*, *in vitro*	2021 [251]
quaternized chitosan (QCS), oxidized dextran (OD), tobramycin (TOB), and polydopamine-coated polypyrrole nanowires (PPY@PDA NWs)	Self-healing	(TOB)	Antibacterial activity against *Pseudomonas aeruginosa, Staphylococcus aureus,* and *Escherichia coli* Have a long antibacterial effect of up to 11 days Enhance wound infection healing *in vivo*	2022 [252]
gelatin methacrylate (GelMA), adenine acrylate (AA), and CuCl2	Self-healing	CuCl2	Show self-healing and adhesive properties Have antibacterial properties promote proinflammatory factors and angiogenesis	2022 [253]
hydroxypropyl chitosan (HPCS) and poly(*N*-isopropylacrylamide) (PNIPAM), dipotassium glycyrrhizinate (DG)	Self-healing	(DG)	Have biocompatibility Reduce inflammatory Have antibacterial activity against *S. aureus* Promote wound healing *in vivo*	2022 [184]
β-Cyclodextrin(β-CD)/polyethyleneimine (PEI)/PVA	Self-healing	(β-CD)	High biocompatibility Antibacterial effect against *S. aureus* and *E. coli*	2022 [254]
Oxidized quaternized guar gum (OQGG)/carboxymethyl chitosan (CMCS)	Self-healing	OQGG@CMCS	Have cytocompatibility ability Exhibit antibacterial and hemostatic properties Promote wound healing	2022 [255]
fusiform-like ZnO (brZnO) and carboxymethyl chitosan (CMCS)	Self-healing	CMCS-brZnO	Reduced the inflammatory ratio Have antibacterial activity against *E. coli* and *S. aureus* Accelerate wound healing	2022 [256]

**Abbreviations:** (AgNPs) Silver nanoparticles; (AA) adenine acrylate; (β-CD) β-Cyclodextrin; (CMCS) carboxymethyl chitosan; (CS DABC) Chitosan dialdehyde bacterial cellulose; (CNF) Cellulose nanofibers; (DG) dipotassium glycyrrhizinate; (OCMCDA) Dopamine-grafted oxidized carboxymethyl cellulose; (GelMA) gelatin methacrylate; (HPCS) hydroxypropyl chitosan; (OD) Oxidized dextran; (OQGG) Oxidized quaternized guar gum; (PVA) Poly(vinyl alcohol); (PEGS-FA) Poly (glycerol sebacate) grafted 4-formylbenzoic acid; (QCSP) Quaternized chitosan polyaniline; (QC) Quaternized chitosan; (VBIMBr) 1-vinyl-3-butylimidazolium; (N, O-CMC/OCS) N, O-carboxymethyl chitosan/oxidized chondroitin sulfate; (NEO) Neomycin; (CB-Dex) Carboxybetaine dextran; (SB-Dex) Sulfobetaine dextran; (CHA) Chlorhexidine acetate; (QP-(DMAPMA)) Quaternized *N*-[3(Dimethylamino) propyl] methacrylamide; (TOB) tobramycin; (PPY@PDA NWs) polydopamine-coated polypyrrole nanowires; (PNIPAM) poly(*N*-isopropylacrylamide); (PEI) polyethyleneimine; (brZnO) fusiform-like ZnO.

### 6.7 Dual-responsive smart antibacterial hydrogels for wound dressing

Some hydrogels that are used in wound healing are dual-sensitive. It means that their releasing activity is in response to two different mentioned mechanisms. Examples include a pH and thermo-sensitive antibacterial hydrogel made from chitosan and PNIPAm loaded with gentamicin sulfate (GS) made by *Qureishi* et al. [257] showed when temperature and pH are elevated, the inhibition zone of *E. coli* is greater. They demonstrated by increasing the temperature drug absorption increased and the zone of inhibition was larger. Also, the polymer chains opened at pH 9.4 and 4.4; thus, the inhibition zone was expanded. *Ma* et al. [258] fabricated a pH- and thermo-sensitive hydroxypropyl chitin/tannic acid/ferric ion (HPCH/TA/Fe) composite hydrogel with antibacterial activity against *E. coli* and *S. aureus* while being able to bypass scar tissue formation in a mouse wound model. Changes in pH and temperature concluded to change in releasing profile of TA and enhanced the bactericidal properties. Gao and colleagues [259] synthesized a thermo-responsive, NIR-controllable, ciprofloxacin-loaded polydopamine NPs and glycol chitosan (Gel-Cip) hydrogel system to eradicate gram-positive and gram-negative bacteria *in vitro*. Additionally, they showed that the combination of NIR and Gel-Cip can improve the healing of infectious wounds in a mouse model.

Qiao et al. [260] designed an intelligent hydrogel that was not only a drug-releasing bactericidal wound dressing via NIR exposure but also a pH-responsive detector for infections. It was composed of silica nanoparticles modified with Cyanin 3 and 5 (SNP-Cy3/Cy5) for the detection of disease in pH alterations as a nanoprobe, PVA, gentamicin sulfate, which has been covalently grafted into PEG via a UV-cleavable linker (GS-Linker-MPEG), and up-conversion nanoparticles (UCNP) to change NIR to UV for the release of GS in the required situation. They concluded that this hydrogel system might be suitable for monitoring infections and an antibacterial wound dressing. Some other dual-responsive hydrogels which possess the potential to alleviate the wound-healing process are listed in Table 7.

**Table 7:** Dual-responsive smart antibacterial hydrogels as wound dressings.

Material(s)	Antibacterial agent(s)	Dual mechanisms	Major finding(s)	Year, ref
Dopamine (DA) and folic acid (FA), Zn2+ (DFT-C/ZnO)	ROS and Zn^2+^	ROS and 660 and 808 ​nm light irradiation	99.9% antibacterial efficacy against *S. aureus* and *E. coli* enhanced the fibroblast growth	2019 [261]
Aldehyde hyaluronic acid (A-HA), adipic acid dihydrazide graft hyaluronic acid (HA-ADH) and sisomicin sulfate (SS) (–A-HA/HA-ADH/SS)	sisomicin sulfate	pH- and HAase- ​responsive	Accelerate healing of full-thickness wounds *in vivo* Antibacterial effect against *S. aureus* and *E. coli*	2020 [262]
phenylboronic acid ​and ​alginate ​and amikacin ​(AM)and naproxen ​(Nap)	Amikacin (AM)	pH- and ​ROS-responsive	90% for ​*S.aureus* ​and 98% for ​*P.aeruginosa in vitro* antibacterial activity perfect anti-inflammatory response and promote wound healing *in vivo*	2020 [263]
phenylboronic acid-modified hyaluronic acid (HA–PBA) and plant-derived polyphenol-tannic acid (TA) and AgNP	AgNP	pH- and ​ROS-responsive	Injectable, cytocompatible (with L929 ​cells) and self-healing hydrogel with antioxidant properties and antibacterial activity against MRSA and *P. aeroginosa in vitro*	2021 [264]
phenylboronic acid and benzaldehyde bifunctional polyethylene glycol-co-poly (glycerol sebacic acid)/dihydrocaffeic acid and ​l-arginine cografted chitosan (PEGS-PBA-BA/CS-DA-LAG and metformin (Met) and graphene oxide (GO) (PC/GO/Met)	metformin (Met) and graphene oxide (GO)	pH and glucose-responsive	reducing inflammation and promoting angiogenesis in a rat type II diabetic foot model antibacterial activity and accelerate wound healing on chronic athletic diabetic wounds	2022 [265]

**Abbreviations:** (AHA) aldehyde hyaluronic acid; (AM) amikacin; (DA) dopamine; (FA) folic acid; (GO) graphene oxide; (HA–PBA) ​phenylboronic acid-modified hyaluronic acid; (Met) Metformin; (Nap) ​naproxen; (SS) sisomicin sulfate; (TA) tannic acid.

### 6.8 Printed smart antibacterial hydrogels as wound dressings

Among several manufacturing methods, 3D bioprinting is particularly beneficial for producing tissue-regenerating scaffolds using a wide range of biocompatible materials. This method is particularly of interest for printed 3D tissue constructs. It provides solid cell-laden freeform scaffolds with tunable mechanical abilities [266]. The dilemma in developing these methods is maintaining the total mechanical integrity and shape constancy of the structures during the printing process or cell culture conditions [267]. In the study Zhao et al. [268]*,* a photoactive cationic conjugated PPV derivative was linked into the 3D printing ink Gel/Alg/HA to produce a conjugated polymer ink Gel/Alg/HA/PPV, which was used to build an artificial skin patch. The cationic PPV induces highly visible light-harvest and better ROS generation properties and superior biocompatibility. Also, in that study, Ca2+ was used as a crosslinker. The electrostatic interaction between anionic Gel/Alg/HA and cationic PPV boosted the formation of an ink system and diminished its toxicity. Gel/Alg/HA/PPV demonstrated antibacterial activities against *S. aureus in vitro* and *in vivo*. This study revealed a direct relationship between the concentration of PPV and bactericidal ability. Subsequently, the antibacterial ability of PPV skin patches was further confirmed in *S. aureus*-infected rat wound models. They performed the experiments on two main groups: PPV skin patch dark and PPV skin patch light. The result of colony count exhibited a wet and suppurative wound 4 days post-treatment in the PPV skin patch dark group versus dry and non-suppurative wounds treated with PPV skin patch light. In another study, the antibacterial activity of cefazolin (CFZ)-containing polycaprolactone 3D scaffold and rifampicin (RFP)-loaded alginate hydrogel encapsulating the 3D scaffold was evaluated by Lee et al. [269] The scaffold displayed a synergistic effect. The RFP inhibited biofilm formation as an external drug, and CFZ enhanced the antibacterial activity as an internal one. The antibacterial properties of the scaffold were assessed using a disk diffusion assay against *S. aureus*. While the CFZ-carrying group exhibited a diameter of inhibition, in the RFP-only group, no inhibitory effects were observed on *S. aureus* growth. The growth rate of *S. aureus* was considerably reduced by the RFP–CFZ group. Some other 3D-printed smart hydrogels showing antibacterial activity in wound healing are listed in Table 8.

**Table 8:** Printed smart antibacterial hydrogels as wound dressings.

Material(s)	Antibacterial agent(s)	Major finding(s)	Year, ref
3D Printed GelMA/Xanthan gum/TiO2/N-halamine dressings (GX2-TiO2-PSPH-Cl)	TiO2	It has an excellent biocompatibility It has a high antibacterial efficiency Possess accelerated wound healing capacity *in vivo*	2021 [270]
3D Printed CMC/PL (CP)/(GMA)/(ε-PL)	CMC/PL	It has an excellent inhibitory effect (95%) on both *E. coli* and *S. aureus* It has been a considerable rise in the expression of VEGF and CD31 Accelerate granulation tissue regeneration	2021 [271]
3D Printed (PNIPAAm)/(ALG), (MC)/(Octenisept®, OCT)	(Octenisept®, OCT)	Has antimicrobial activity against *S. aureus*, *Candida albicans*, and *P. aeruginosa* Has non-cytotoxicity towards fibroblasts	2021 [272]
3D Printed ZnO nanoparticles modified PVDF/(SA)/(ZPFSA)	ZnO	Exhibits good biocompatibility Excellent antimicrobial properties The stable piezoelectric response can remarkably boost the wound healing	2022 [273]
The outer layer PCL/PLA (PP)/the inner layer 3D printed by optimized SA/PVA/chitosan quaternary ammonium salt (SPH)	vancomycin (VCM)	A bilayer asymmetric was enhanced to mimic the gradient structure of the epidermis and dermis It has antibacterial activity against *S. aureus* It has no significant cytotoxic effects on (HFBS)	2022 [268]
3D Printed AgNPs were synthesized using banana peels	(AgNPs)	Show antibacterial activity against Gram-negative and Gram-positive bacteria It has swelling and consistency properties	2022 [274]

**Abbreviations:** (ALG) sodium alginate; (CMC) carboxymethyl cellulose; (GMA) glycidyl methacrylate; (HFBS) human dermal fibroblasts; (MC) methylcellulose; (Octenisept®, OCT) octenidine dihydrochloride and 2-phenoxyethanol; (ε-PL) ε-polylysine; (PNIPAAm) poly(*N*-isopropylacrylamide); (SA) sodium alginate; (VCM) vancomycin; (ZPFSA) piezoelectric hydrogel scaffold.

### 6.9 Antibacterial smart hydrogels under clinical investigations or in the market

Over the years, various wound dressings have been commercially available for wound repair approaches through cutting-edge technologies. Because of this, recent skin wound dressing products have increased the survival rates of burn and trauma patients. Hydrogels have also shown outstanding merits in medical applications thanks to their unique properties and reversible supramolecular interactions and their capacity to include numerous therapeutic agents as new drug delivery systems to achieve effective treatments [275]. Noorbala and colleagues [73] assess and compare the effectiveness of Iranian hydrogel sheet (Irgel) produced by radiation and MaxGel. In this study, participants included 90 patients with second-degree burn injuries who were presented to the Yazd Burn hospital and were randomized into three equal groups: sterile vaseline gauze (negative control), an Iranian hydrogel sheet (Irgel) (test group), and MaxGel (positive control group). The burns and pain sensations were quite similar among the different groups before the intervention. In contrast, the burn size and pain intensity for the Irgel group were considerably smaller than those of the standard control and the positive control groups after a week. At a hospital located in Brisbane, Australia, Nilforoushzadeh et al. [276] evaluated the effectiveness of the novel prevascularized skin grafts containing the dermal and epidermal layer using the adipose stromal vascular fraction (SVF)-derived endothelial cell on 10 patients with diabetic wounds compared with nonvascularized skin grafts as the control. The result showed that skin thickness and density in the vascular beds treated with the hypodermis autologous dermal-epidermal skin grafts with intrinsic vascular plexus was remarkably higher than the control group. Table 9 and Table 10 depicts promising results in healing different types of human skin wounds, while Table 11 shows commercially available dressings products on the market today.

**Table 9:** Different wound dressing hydrogels under clinical trials.

Product(s)	Treatment procedure	Number of patients	Period follow up	Country	Significant finding(s)	Year, ref
SilvaSorb® Gel	Control group treatment with Silvadene® silver sulfadiazine cream, a randomized group with treatment with Silva Sorb Gel	24 patients	Day 1 and every 2–3 days for up to 21 days	Philadelphia, Pennsylvania (USA)	The SilvaSorb Gel boosts re- epithelialization and reduces pain Reduces infection	2009 [277]
Human fibroblast growth factor 2 FGF-2 (rhFGF-2) in a gelatin hydrogel	Randomly assigned to three groups receiving a single injection of the gelatin hydrogel containing either placebo or 0.8 ​mg (low-dosage group) or 2.4 ​mg (high-dosage group) of rhFGF-2	194 Patients	24-week	Institutions in Japan	The percentages of patients with the radiographic bone union were higher in the rhFGF-2-treated groups (p ​= ​0.031 and 0.009 in low- and high-dosage groups, respectively) compared with the placebo group, although there was no significant difference between low- and high-dosage groups.	2010 [278]
Protease-modulating polyacrylate- (PA-) containing hydrogel	Control group treatment with amorphous hydrogel, second group treatment with PA-based hydrogel	75 patients	Two weeks wound assessment on days 7 and 14	France	The PA-based hydrogel group showed a considerable reduction in fibrin and necrotic tissue PA-based hydrogel group increases the area covered by granulation tissue	2014 [279]
Punica granatum L. Hydrogel for Wound Care Treatment	2% (w/w) Punica granatum peel ethanolic extract (PGMF) based on a hydrophilic cream and zinc oxide, Oral iron therapy	A 76-year-old woman	Six weeks	Brazil	The ulcer had decreased to one-quarter of its original size and had healed entirely six weeks later. No adverse effects of PGMF were observed.	2016 [280]
Oleogel-S10	Control group treatment with Octenilin®, randomized group treatment with Oleogel-S10	61 patients	Wound dressings were changed every two days at 12 months	Ten centers in 4 countries (Germany, n ​= ​4; Sweden, n ​= ​2; Switzerland, n ​= ​1; UK, n ​= ​3)	Oleogel-S10 has a higher antibacterial effect against Gram-positives and Gram-negatives; Increased the percentage of epithelialization; Oleogel-S10 reduced the texture, pigmentation, and redness of the skin	2018 [281]
Chitosan Gel	The tolerability and efficacy of chitosan gel formulation were determined	20 patients	30 days (Dressing change twice a week)	Italy	Treatment was effective Increase the wound healing rate	2018 [282]

**Abbreviations:** (rhFGF-2) Human fibroblast growth factor 2 FGF-2; (PA) polyacrylate; (PGMF) Punica granatum peel ethanolic extract.

**Table 10:** Different wound dressing hydrogels under clinical trials.

Status	Study Title	Conditions	Interventions	Locations	Year, ref
Completed	Modified Surface of PLGA/Nanoparticles in Smart Hydrogel	Antibiotic-Resistant Infection	Drug: Ciprofloxacin	Mona Arafa Cairo, Egypt	2022 NCT05442736
Completed	Topical Erythropoietin Hydrogel Formulation for Diabetic Foot Ulcers	Diabetic Foot Ulcer	Drug: A hydrogel containing erythropoietin Drug: Hydrogel (as a part of SOC)	HaEmek and Edith Wolfson Medical Center Afula, Haifa, Holon Israel Rambam Health Care Campus	2022 NCT02361931
Completed	Safety Study of Topical Doxycycline Gel for Adult Diabetic Lower Extremity Ulcers	Diabetic Foot Ulcer	Drug: doxycycline Drug: placebo gel	North Florida/South Georgia Veterans Administration Hospital Gainesville, Florida, United States	2010 NCT00764361
Completed	Efficacy of My Skin Patch for the Healing of Cut Injuries and Abrasions	Wounds and Injuries Abrasion Cut Injuries	Device: My Skin patch Device: Gauze and Patch	Ospedale Bellaria UOC Dermatologia Bologna, Italy	2014 NCT01573234
Completed	Efficacy and Safety of Loxoprofen Hydrogel Patch in Patients with Ankylosing Spondylitis	Ankylosing Spondylitis	Drug: Loxoprofen sodium hydrogel patch Drug: Loxoprofen sodium tablet	Department of Rheumatology at the Third Affiliated Hospital of Sun Yat-sen University Guangzhou, Guangdong, China	2019 NCT03800797
Recruiting	Effect of the Negative Pressure Therapy Dressing Compared with Hydrogel Dressing.	Wound Infection, Surgical	Procedure: Pico®" negative pressure dressing Procedure: Aquacel Surgical®" hydrogel dressing	Pilar Garrido Martín Santa Cruz De Tenerife, Spain	2021 NCT04265612
Recruiting	Quantitative and Clinical Assessment of Flexor Tendon Gliding Following Application of a Bioresorbable Hydrogel: A Prospective, Randomized Study in Patients Undergoing Distal Radius Fracture Repair	Distal Radius Fracture Tendon Rupture	Device: Versawrap membrane	1. University of Colorado Health Hospital Aurora, Colorado, United States 2. Denver Health Hospital Denver, Colorado, United States	2021 NCT04976335
Completed	A Prospective, Descriptive Cohort Study with Prontosan® Wound Gel X in Partial and Full Thickness Burns Requiring Split Thickness Skin Grafts	Burns	Device: Prontosan Wound Gel X	Berufsgenossenschaftliche Unfallklinik Ludwigshafen, Baden-Württemberg, Germany Berufsgenossenschaftliches Unfallklinikum Bergmannsheil Bochum, Germany	2018 NCT01534858
Completed	Wound Management for Sacral Pressure Ulcers with Necrotic Tissue	Pressure Ulcers	Device: TheraHoney HD Device: Skintegrity	Swedish Covenant Hospital Chicago, Illinois, United States Harmony Residential Facility Chicago, Illinois, United States	2018 NCT02224638
Completed	Open-Label Test of the HM242 Medical Devices to Evaluate Safety and Local Tolerability	Intact Skin	Device: HM242	Dermatologische Praxis Prager & Partner Hamburg, Germany	2020 NCT04192123
Recruiting	Hidradenitis Suppurativa (HS) Tunneling Wounds	Hidradenitis Suppurativa	Device: antibiofilm surfactant wound gel (ABWG)	University of Miami, Florida, United States	2022 NCT04648631

**Abbreviations:** (ABWG) antibiofilm surfactant wound gel; (HS) Hidradenitis Suppurativa; (PLGA) Poly (lactic-co-glycolic acid).

**Table 11:** List of commercially available dressings based on hydrogels.

Products	Company	Features
3 ​M™ Kerralite Cool™ Hydrogel Dressing	3 ​M Health Care	It can be used on contaminated and colonized wounds Used for the management of chronic wounds
GEMCORE360°™ Hydrogel Dressing	GEMCO Medical	Can treat different types of wounds Provides excellent fluid donation
Energel™ Wound Hydrogel	Vomaris Wound Care	Can reduce infection
Derma-Gel® Hydrogel Sheet	Medline Industries	It can be utilized for various types of wounds Manages bacterial burden and protects wounds from bacteria It helps create a moist wound environment to promote healing
Avogel® Hydrogel Sheeting	Avocet Polymer Technologies	Controls scar formation to enhance skin function Bacteria or fluid barrier potentiality
AquaDerm™ Hydrogel Sheet	DermaRite Industries	Used for partial- and full-thickness wounds Bacteria or fluid barrier potentiality Supports moist wound healing
AMERIGEL® Hydrogel	AMERX Health Care	Used for wound infection Sustains moisture longer than standard hydrogels Ease of application and use increases patient compliance
Viniferamine® Wound Hydrogel Ag	Viniferamine	High-viscosity, an enduring hydrogel providing a bed with the moisture required to smooth cell migration Stabilizes the pH, temperature, and wound hydration Can reduce infection
Suprasorb® G Hydrogel Sheet Dressing	L&R USA	Initial cooling and ongoing soothing wound pain relief Color change indicates when it is time to change the dressing Bacteria or fluid barrier potentiality
SilverMed™ Amorphous Hydrogel	MPM Medical	Sustained release of microbial silver ions that makes a long-lasting antimicrobial barrier Continues to provide antimicrobial effectiveness up to six days Contains no sulfonamides
Pure&Clean Antimicrobial Hydrogel	Pure&Clean	Adds moisture to dry wound beds Relieves itching and pain associated with dermal irritations Antibacterial activity
Puracyn® Plus Professional Formula Antimicrobial Hydrogel	Innovacyn	Non-toxic and non-irritating Can reduce infection
MEDIHONEY® Hydrogel Sheet Dressing (Non-Adhesive)	Integra LifeSciences	A dressing will form a gel as it warms up and contacts wound fluid Useable on infected wounds
McKesson Hydrogel Sheet Dressing	McKesson Medical-Surgical	Provides a wet wound-healing environment It helps boost autolytic debridement Useable on infected wounds

## 7 Other types of antibacterial hydrogels as wound dressings

Antibacterial hydrogels made of polymers with inherent antibacterial activity have been developed to prevent wound infection. For instance, Gan et al. [55] fabricated an antimicrobial mussel-inspired contact-active polyampholyte hydrogel. To this end, they used quaternized chitosan (QCS) and AA-co-MADA-co-DMAEMA (AMD) as a physical cross-linking generated from methacrylamide dopamine (MADA), 2-(dimethylamino) ethyl methacrylate (DMAEMA) used as a chemical cross-linker to fabricate the hydrogel. Based on their study, QCS and AMD displayed a synergistic antimicrobial effect against Gram-positive and Gram-negative bacteria. The AMD-QCS hydrogel antibacterial study on *E. coli* and *S. epidermidis* showed that the bactericidal effects of AD and AMD hydrogels on both strains were 23% and 44%, respectively. In comparison, AD-QCS and AMD-QCS hydrogels showed much higher antibacterial rates (70% & 87%, respectively). The antibacterial activity of AMD-QCS was also tested against *E. coli*-infected Sprague Dawley (SD) rats. Notably, the hydrogel group effectively killed the bacteria and almost sealed the wound in comparison with the control.

Hydrogels based on silver-ethylene PAM/hydroxypropyl methylcellulose (HPMC) have also shown good antibacterial properties as antibacterial dressing hydrogels. For example, the disk diffusion assay determined the inhibition zone of AgNP-loaded super porous hydrogel against *S. aureus* and *E. coli* with promising results. Chitosan and HPMC-containing hydrogels were used as control groups, and the results indicated no antibacterial activity of chitosan/HPMC hydrogel against both strains. Simultaneously, the AgNP hydrogel revealed an inhibition zone against *S. aureus* (1.25 ​mm) and *E. coli* (1.12 ​mm). However, the antibacterial ratio for AgNPs hydrogel was 86.5% and 75.8% against *S. aureus* and *E. coli*, respectively. The antibacterial property of AgNPs hydrogel against *S. aureus* was also studied in the wound infection created on the SD rat model. The result showed no infection prevention in HPMC and chitosan-containing samples, while AgNPs hydrogel exhibited good antibacterial activity, prevented infection, and promoted wound healing [283].

There are also studies on biological antibacterial agent-loaded hydrogels as wound dressings. For example, Bagher and coworkers [284], synthesized an Alginate/Chitosan hydrogel loaded with Hesperidin. Hesperidin is a flavonoid extracted from vegetables and fruits that possess antibacterial properties. The antibacterial activity of hydrogel with different concentrations of hesperidin (1% and 10%) was examined against *S. aureus* and *P. aeruginosa*. Alginate/Chitosan hydrogel, as the control group, demonstrated low antibacterial activity. Therefore, the hydrogel with 10% hesperidin exhibited the most antibacterial activity, with significantly fewer colonies in both strains.

Hydrogels as a drug delivery system have been used to sustain the release of antibiotics in low concentrations at the target site and enhance antibacterial efficiency [285]. Multilayer hydrogels also indicate promising properties for a potential antibacterial agent-releasing system [286]. For example, a hydrogel with four layers of PVA, gelatin, ampicillin-loaded HA, and gelatin showed great potential for the sustained release of ampicillin. The antibacterial behavior of ampicillin disk (10 μg/disc), ampicillin-contained multilayer hydrogel (ML-D), and multilayer hydrogel with no ampicillin (ML) were evaluated against oxacillin-sensitive *S. aureus*, oxacillin-resistant *S. aureus*, and ampicillin-resistant *E. coli*. The zone of inhibition (ZOI) for ampicillin, ML, and ML-D against oxacillin-sensitive *S. aureus* were 19 ​± ​1.0 ​mm, 14 ​± ​2.0 ​mm, and no inhibition zone, respectively. It is worth mentioning that no inhibition zone was observed for oxacillin-resistant *S. aureus* and ampicillin-resistant *E. coli* among all experimental groups. Raj and coworkers evaluated the antibacterial activity of collagen mimetic peptide (CMP) tethered vancomycin (Van)-containing liposomes (Lipo) (CMP-Van-Lipo) hybridized against methicillin-resistant *Staphylococcus aureus* (MRSA) at the infected wound site both *in vitro* and *in vivo*. CMP-Van-Lipo nanostructures were able to release the Van sustainably and increased the antibacterial activity against MRSA, compared to Van-Lipo-loaded co-gels or Van-loaded co-gels. This activity was shown to be dose-dependent. The *in vivo* results revealed that the growth of bacteria was entirely reduced in CMP-Van-Lipo-loaded co-gels compared to Van-Lipo-loaded co-gels [36].

Several studies confirm the antibacterial effects of copper nanoparticles (Cu NPs) against Gram-positive and Gram-negative strains [287]. Cu NPs-loaded double-layer electrospun PVA, chitosan, and PVP showed a sustained release of copper with antibacterial properties against *S. aureus*, *P. aeruginosa*, *E. coli*, and *Bacillus cereus*. Claus and coworkers [288] polymerized ionic liquids-based hydrogels using 11 types of polymerized ionic liquids (PILs). The time-kill assay evaluated the antibacterial activity of different monomers without any antibacterial agent-loaded against *S. aureus* and *P. aeruginosa*. The test was performed based on four main groups of polymers: cationic, anionic, neutral, and zwitterionic. The result exhibited that all cationic-based polymers possessed significant inherent antibacterial properties against both strains (>70%). Furthermore, the imidazolium hydrogels showed a high-killing efficiency (around 95%), which confirmed the antibacterial activity of PILs-based hydrogels (Fig. 8C). Mainly, the highest inhibition in bacterial growth was attributed to poly (VBImCl) and poly (VBImBr) even in the least volume of polymerized hydrogel (25 ​μl). The data interpretation revealed that anionic-, neutral-, and zwitterionic-based polymers demonstrated a more extraordinary antibacterial ability against *S. aureus* (MRSA) than *P. aeruginosa*. This could be due to the various cell membrane structure of gram-negative and gram-positive strains. As indicated in (Fig. 8D), the monomers based on imidazolium, such as VBImBr, VBImCl, HEMA, and TMA-VB, can maintain at least 87% of their antibacterial activity over one week. Table 12 reviews recent studies on other antibacterial hydrogels between 2019 and 2022.

**Table 12:** Other different types of antibacterial hydrogels.

Material(s)	Antibacterial agent(s)	Major finding(s)	Year, ref
OHA-AT/CEC	Amoxicillin	Excellent anti-oxidant Cell compatibility on C2C12 ​cells Antibacterial properties against *E. coli and S. aureus* Accelerated wound healing	2019 [289]
Poly (ionic liquid)/PVA	Poly (ionic liquid)	High mechanical strength Antibacterial activity can kill *S. aureus*, *E. coli*, *and B. subtilis* Biocompatibility on MC3T3-E1 cells Promotes wound healing	2019 [290]
QCS-MPAM	QCS	Excellent mechanical strength Biocompatibility on L929 Antibacterial effect against *E. coli*, *S. epidermidis*, *and S. aureus* Promotes wound healing	2019 [291]
Gel-MA/BACA/Cu	Cu NPs	No cytotoxicity for NIH-3T3 fibroblast Antibacterial properties against *E. coli* and *S. aureus* Promotes tissue regeneration	2019 [13]
Hydrogel-based PVP/PVA/PN	PN	Highest swelling rate High antibacterial ability against *E. coli* and *S. aureus*	2019 [292]
Hydrogels incorporating CHG	CHG	Antibacterial properties against *E. coli* and *S. aureus* Non-toxic Promotes wound healing	2020 [293]
Hydrogels loaded with heparinized ZnO NPs	ZnO NPs	Biocompatibility on L-929 and HDF cells Antibacterial effect against *E. coli* and *S. aureus* Enhanced re-epithelialization and collagen formation *in vivo*	2020 [294]
Hydrogel based on chitosan/hyaluronan/PCDQ	PCDQ	Antibacterial properties against *E. coli*, *K. pneumoniae*, *P. aeruginosa*, *S. aureus*, and *S. haemolyticus* Non-toxic on fibroblast cells (NIH 3T3) Enhanced re-epithelialization	2021 [295]
Hydrogel-based ADH/γ-PGA/(KGM)	γ-PGA/OKGM	Antibacterial ability against *E. coli* and *S. aureus* Promote inflammatory response in rats Enhance wound healing	2021 [296]
polyethylene glycol monomethyl ether Modified glycidyl methacrylate functionalized chitosan (CSG-PEG), methacrylamide dopamine (DMA) and zinc	zinc	Have excellent inherent antibacterial activity against Methicillin-resistant *S. aureus* (MRSA) Promote the expression of CD68 and CD31 Enhance wound infection healing *in vivo*	2022 [297]
Oxidized alginate (ADA) and catechol-modified gelatin (Gel-Cat) polydopamine decorated silver nanoparticles (PDA@AgNPs)	(PDA@AgNPs)	Have antibacterial properties against *S. aureus* and *E. coli in vitro* Reduce wound infection *in vivo* Enhance angiogenesis	2022 [298]
Gelatin/iron- based metal-organic framework nanocomposite/Camellia sinensis (CS)	(CS)	Water absorption was increased due to the interaction of iron-based metal-organic framework within the hydrogel matrix Show antibacterial property against “*Bacillus serous”, “S. aureus”, “S. mutans"," E. coli”, “Klebsiella pneumoniae”,* and *“P. aeruginosa*"	2022 [299]
2,2,6,6-tetramethylpiperidine-1-oxyl (TEMPO)-doped multifunctional/quaternized carboxymethyl chitosan (QCMCS) and oxidized hyaluronate (HA-CHO)	Quaternary ammonium	Have a rheological property Exhibit inherent antibacterial and antioxidant properties Have excellent cytocompatibility Reduce the level of hemolysis	2022 [300]
polyethyleneimine (PEI), polyethylene glycol (PEG), hexachlorocyclic triphosphonitrile (HCCP), and gold nanoparticles	gold nanoparticles	Antibacterial effect against MRSA and *S. aureus* Show antioxidative activity	2022 [301]

**Abbreviations:** (ADA) Oxidized alginate; (ADH) adipic acid dihydrazide; (CS) Camellia sinensis; (CSG-PEG); polyethylene glycol monomethyl ether modified glycidyl methacrylate functionalized chitosan; (CHG) Chlorhexidine gluconate; (DMA) methacrylamide dopamine; (Gel-Cat) catechol-modified gelatin; (HA-CHO) oxidized hyaluronate; (HCCP) hexachlorocyclic triphosphonitrile; (Gel-MA/BACA/Cu) Methacrylate modified gelatin and N, *N*-bis(acryloyl)cystamine-chelated Cu nanoparticles; (KGM) Konjac glucomannan; (OKGM) oxidized Konjac glucomannan; (PN) Pomegranate; (PCDQ) Phosphatidylcholine dihydroquercetin; (PDA@AgNPs); polydopamine decorated silver nanoparticles; (γ-PGA) γ-polyglutamic acid; (PEI) polyethylenimine; (PEG) polyethylene glycol; (QCS-M-PAM) Quaternized chitosan-Matrigel-polyacrylamide; (QCMCS) multifunctional/quaternized carboxymethyl chitosan; (TEMPO) 2,2,6,6-tetramethylpiperidine-1-oxyl-doped.

## 8 Remarks and future prospective

Wound infection is one of the main concerns in healthcare systems. The traditional route and overuse of antibiotics have caused increased antibiotic resistance. The high prevalence of this phenomenon leads to drug inefficiencies and increases multidrug-resistant bacteria. Therefore, it is essential to design novel drug delivery systems that can control the release of drugs within the target site and thus reduce the speed of antibiotic resistance. In addition, these organised systems should be able to accelerate the wound healing process to repair the body's first line of defense quickly. Thus, itself diminishes the risk of further infection and also the emergence of new antibiotic resistant strains. Smart hydrogels with inherent antibacterial activities or loaded with antibacterial agents are introduced as an alternative for overcoming this dilemma. Smart antimicrobial hydrogels are promising as they sustainably release the drugs to promote antibacterial activity and prevent the risk of infections. Also, in this way, the side effects caused by antibiotics are reduced by the gradual release of antibiotics on the target site. Hence, intelligent, antibacterial hydrogels are appropriate for preventing wound infection, especially against multi-drug-resistant bacteria. In this comprehensive review, different fabrication strategies of smart hydrogels are introduced, several materials are recommended, and many studies are summarized to represent current trends regarding this urgent global concern. Many introduced biomaterials and fabrication techniques here can shed light on future trends.

## Declaration of competing interest

The authors declare that they have no known competing financial interests or personal relationships that could have appeared to influence the work reported in this paper.

## Data Availability

Data will be made available on request.
